# Differences in Microbial Community Succession and Caproic Acid Metabolic Dynamics in Upper-Layer Fermenting Grains Between Aged and New Pits During Strong-Flavor Baijiu Fermentation

**DOI:** 10.3390/foods15213975

**Published:** 2026-11-05

**Authors:** Huimin Zhang, Kangjie Xu, Qiang Chang, Li Zhen, Yongqing Li, Tianwen Wang, Bo Wang, Yan Liu

**Affiliations:** 1 College of Biological and Food Engineering, Anhui Polytechnic University, Wuhu 241000, China; xukangjie2025@126.com (K.X.); antangelaine@163.com (L.Z.); wangtw@mail.ahpu.edu.cn (T.W.); wbhg13956338589@126.com (B.W.); 2 Anhui Jinyuwan Distillery Co., Ltd., Fuyang 236500, China; m18726505225_1@163.com (Q.C.); l13135581191@icloud.com (Y.L.)

**Keywords:** fermenting grains, amplicon sequencing, caproic acid biosynthesis and esterification, co-occurrence network, lactic acid bacteria, synthetic microbial community

## Abstract

Caproic acid and its ester ethyl caproate are the characteristic flavor compounds of Chinese strong-flavor Baijiu (CSFB), yet their synthesis and esterification in upper-layer fermenting grains (UFG), and how these processes differ between new and aged pits, remain unclear. Compared with aged-pit UFG, the physicochemical parameters, microbial communities, and metabolite dynamics of new-pit UFG likewise followed a largely similar three-stage pattern: early acidification and starch consumption (2–8 d), mid-stage stabilization (10–20 d), and late secondary acid/ester synthesis (30–60 d). The critical divergence lay in butyric/caproic acid synthesis and the eukaryotic community: new-pit UFG produced lower amounts of butyric and caproic acids, and was dominated by taxa of unclear fermentative relevance, lacking the non-Saccharomyces yeasts (*Kazachstania*, *Candida*) enriched in aged-pit UFG, whose abundances correlated with ester accumulation. Specifically, new-pit UFG yielded lower concentrations of three of the four major ethyl esters, whereas ethyl lactate was consistently higher than in aged-pit UFG; yet, notably, ethyl butyrate accumulated despite nearly undetectable butyric acid. Correlation analyses identified candidate caproic acid producers (*Lactobacillus*, *Pediococcus*, *Weissella*) and esterification contributors (*Candida*, *Mucor*). These findings highlight the importance of the predicted maturity-driven metabolic–ecological network integrity in UFG, suggesting potential for synthetic-microbiome strategies in new-pit fermentation.

## 1. Introduction

Solid-state fermentation of Chinese strong-flavor Baijiu (CSFB) is a complex process driven by a multi-species microbial consortium, which simultaneously carries out saccharification and alcoholic fermentation while generating diverse flavor compounds. Among these, caproic acid and its ester ethyl caproate are the characteristic flavor compounds of CSFB and are both specified in the current national standard (GB/T 10781.1-2021) [1]. Therefore, understanding the microorganisms that drive caproic acid synthesis and esterification is crucial to elucidating CSFB quality formation.

Considerable progress has been made in recent years in elucidating caproic acid synthesis and its esterification during CSFB fermentation. Pit age is a key determinant of caproic acid production: it shapes the microbial community of pit mud, which in turn influences the fermenting-grain (FG) microbiota and ultimately determines fermentation outcomes [2,3,4]. Accordingly, inoculation of pit mud with caproic acid-producing bacteria (CPBs) or their consortia has been attempted in efforts to enhance fermentation performance [5,6,7]. Existing studies indicate that CPBs are mainly enriched in the surface layer of bottom pit mud [2]. Culture-independent analyses based on 16S rRNA gene amplicon sequencing (Silva database v138) have identified *Caproiciproducens* (family Oscillospiraceae) as the dominant genus of the CPB community [5,8], and culture-dependent studies have isolated CPB strains affiliated predominantly with Oscillospiraceae (genera *Caproicibacterium* and *Caproicibacter* [9,10,11,12,13,14]) as well as non-Oscillospiraceae taxa such as *Clostridium*, *Enterococcus*, and *Rummeliibacillus* [15,16,17]. These CPBs are generally thought to migrate from pit mud into the FG–Huangshui microsystem during the late fermentation stage, where they drive caproic acid synthesis through interactions between pit mud and FG [18,19,20]. Beyond caproic acid itself, its esterified product ethyl caproate—together with the esters of its biosynthetic precursors and intermediates, namely ethyl lactate, ethyl acetate, and ethyl butyrate—constitutes the major ester fraction that defines the aroma profile of CSFB [21,22]. In the FG, esterification is driven primarily by the eukaryotic community, including yeasts and filamentous fungi, as well as lactic acid bacteria (LAB) [23,24,25]. Notably, esterification is metabolically coupled to caproic acid synthesis: the sequestration of caproic and butyric acids into their ethyl esters not only shapes aroma quality but also alters substrate and product availability, thereby feeding back on chain elongation. A comprehensive understanding of caproic acid metabolism in the FG therefore requires simultaneous consideration of both synthesis and esterification, and of eukaryotic communities alongside prokaryotes.

However, the pit mud microbiota is spatially stratified: pit-wall and pit-bottom mud differ markedly, with the latter harboring higher CPB abundances and greater metabolic potential [26,27,28]. Correspondingly, the diversity of known CPBs and the concentrations of fatty acids (e.g., butyric and caproic acids) and their esters in FG increase with depth: the bottom-layer FG contains significantly higher levels than the upper layer [29,30,31,32]. This vertical heterogeneity arises from the material-transfer gradient across the FG–Huangshui–pit-mud ecosystem. The lower-layer FG, positioned below the Huangshui line and adjacent to bottom pit mud, receives gravity-driven percolation of Huangshui and microbes from the upper layers and provides a favorable enrichment environment for pit mud-derived functional microbes, including CPBs [18,19,20]. In contrast, the upper fermenting grains (UFG), located above the Huangshui line, is distant from pit-mud-source CPBs, and mass-transfer limitations restrict microbial migration from pit mud; consequently, traditional CPBs fail to colonize the UFG effectively. The UFG thus represents a more suitable model for probing the intrinsic caproic-acid-producing potential of the in situ FG microbiota. Indeed, traditional CPB genera (*Caproiciproducens*, *Caproicibacterium*, *Caproicibacter*, and *Clostridium*) are scarce or even undetectable in the UFG, in marked contrast to the bottom-layer FG, which harbors significantly higher levels of caproic acid and its biosynthetic precursor butyric acid, as well as their corresponding esters [29,30,31,32]. Moreover, known CPBs generally prefer weakly acidic or neutral conditions [9,10,11,12,13,14,15,16,17,18,19,20,26], whereas the FG is a low-pH, high-ethanol system containing inhibitory concentrations of lactic acid and ethanol [29,30,31,32,33]. New and aged pits differ markedly in fermentation quality [4,34]; however, a comparative analysis of CPB community succession and caproic acid metabolic dynamics as well as the associated esterification potential in the UFG of new versus aged pits—previous studies having applied non-targeted profiling to survey the overall flavor composition, without targeted quantification of the dynamics of caproic-acid-related acids and esters on a concentration scale—is still lacking. Such knowledge is critical for designing CPB inoculation strategies targeting newly built pits, particularly because potential CPBs ultimately function within their in situ habitat, the FG, whose upper layer—being minimally influenced by pit-mud-derived CPBs—offers a favorable window into this in situ synthesis metabolism.

Accordingly, this study compares the UFG of new pits with that of aged pits as the reference, through a high-resolution time-series analysis of prokaryotic and eukaryotic community succession, physicochemical parameters, and caproic-acid-related metabolites throughout fermentation, complemented by PICRUSt2-based functional prediction and genus-level correlation analyses. The objective of this study is to investigate the microbial and environmental factors associated with the differences in caproic acid synthesis and esterification between new- and aged-pit UFG, thereby identifying candidate functional genera as potential targets for synthetic microbiome engineering aimed at improving fermentation outcomes in new pits.

## 2. Materials and Methods

### 2.1. UFG Samples Collection

The UFG samples were collected from the pits of a well-known CSFB distillery in Northern Anhui Province, China. The pits measured 2.5 m × 1.5 m × 2 m (length × width × depth), with a normal fermentation period of 60 days. To compare the microbial community and metabolic dynamics between different pit ages, three aged/old pits (age > 40 years) and three newly built pits (age < 3 years; hereafter referred to as new pits) under normal production conditions were selected. UFG samples were collected on days 0, 2, 4, 6, 8, 10, 15, 20, 30, 40, 50, and 60 of a single 60-day batch fermentation (mid-September to mid-November 2024) using a specially designed sampling tool, with each sample taken from the layer between the Huangshui line and the covering FG layer (Figure S1; Table S2). Given that FG microbial communities undergo drastic reconstruction during the early stage before stabilizing [29,33,35], sampling was intensified at 2-day intervals during days 0–10 to resolve this rapid succession, with a transitional phase on days 15 and 20, whereas the stabilized later stage was covered at 10-day intervals. In total, samples were collected at days 0, 2, 4, 6, 8, 10, 15, 20, 30, 40, 50, and 60 of fermentation (12 time points). At each time point, three sub-samples were taken from each pit and mixed in equal proportions to obtain a representative sample. It should be noted that the prokaryotic community data of the aged-pit UFG samples were adopted directly from a parallel investigation [36]; in this study, the eukaryotic community of these samples was further detected, and all subsequent comparative and correlation analyses were performed here. In this comparative framework, the aged-pit UFG serves solely as the reference, whereas the new-pit UFG—for which both the prokaryotic and eukaryotic communities were fully characterized—is the focal system of this study. All UFG samples were coded by pit type, sampling day, and individual pit. For example, “N_15d_1” denotes the composite UFG sample collected on day 15 from new pit 1—one of the three replicate new pits—where “N” and “O” indicate new and aged pits, “15d” the sampling day, and the trailing number the individual pit.

### 2.2. DNA Extraction, Amplicon Sequencing, and Bioinformatics

Total DNA was extracted from the new-pit UFG samples using an E.Z.N.A. soil Kit (D5625-01, Omega Bio-Tek, Norcross, GA, USA) according to the manufacturer’s instructions. The 16S rRNA gene amplicon sequencing data of the aged-pit UFG samples were adopted directly from a parallel investigation [36]. In this study, to ensure comparability, the V3–V4 region of the 16S rRNA gene was amplified for the new-pit samples using the same primer set (338F/806R) and PCR protocol as in [36]. For both aged- and new-pit UFG samples, the ITS1 region of the internal transcribed spacer (ITS) was amplified with primers ITS5F (5′-GGAAGTAAAAGTCGTAACAAGG-3′) and ITS1R (5′-GCTGCGTTCTTCATCGATGC-3′). Amplicons were assessed for quality and quantified, followed by high-throughput sequencing on a NovaSeq 6000 platform by Shanghai Personal Biotechnology Co., Ltd. (Shanghai, China).

After sequencing, raw reads of both new- and aged-pit samples were quality-filtered and processed using QIIME 2 (version 2022.11). Primer sequences were trimmed, and paired-end reads were merged, denoised, and resolved into amplicon sequence variants (ASVs) using the DADA2 plugin. To ensure methodological consistency and cross-pit comparability, the 16S rRNA gene sequencing data from both pit types were re-processed de novo from the raw reads using identical DADA2 parameters and re-analyzed together to generate a unified prokaryotic ASV table. Taxonomic annotation of prokaryotic ASVs was performed against the SILVA database (version 138), in which known CPBs are classified within the genus *Caproiciproducens* [2,5,8]; ASVs assigned to this genus were accordingly treated as traditional CPBs. Mitochondrial and chloroplast sequences that were not completely removed at the experimental stage were further filtered from the samples; after annotation, these sequences were manually removed before subsequent data analysis. For alpha-diversity analysis, all samples were rarefied to the same number of sequences, whereas for other downstream analyses, sequences were used without rarefaction [37]. The ITS1 data were processed as described above to generate a unified eukaryotic ASV table, and eukaryotic ASVs were taxonomically annotated against the UNITE database (version 9.0). The two ASV tables were rarefied to an even sequencing depth for downstream analyses of community composition and α- and β-diversity. Principal component analysis (PCA) was performed separately on the two ASV tables (aggregated to the genus level based on taxonomic annotation) to evaluate between-sample similarity. The potential metabolic functions of the prokaryotic and eukaryotic microbiota were predicted using PICRUSt2 (Phylogenetic Investigation of Communities by Reconstruction of Unobserved States) based on the KEGG (Kyoto Encyclopedia of Genes and Genomes) and MetaCyc databases for prokaryotes and eukaryotes, respectively [38,39].

### 2.3. Detection of Physicochemical Properties

For physicochemical property analysis, the potential substrates (glucose, lactic acid, and acetic acid), intermediate product (butyric acid), and final product (caproic acid) of CPBs, as well as the physicochemical parameters affecting CPB growth (ethanol, pH, NH_4_^+^-N, and temperature) were determined. All physicochemical parameters of the new-pit UFG samples were determined in this study. For the aged-pit UFG samples, all parameters except the four esters were adopted directly from a parallel investigation [36], whereas the four major esters were quantified in this study. The methods are described below. The in situ temperature of UFG during fermentation was monitored using a digital temperature sensor. Starch and NH_4_^+^-N contents were analyzed according to Shen [40]. Ethanol content in solid-state UFG was determined after extraction following the method described by Zhang et al. [41]. Moisture was measured by oven drying at 105 °C to constant weight. The pH of fresh UFG was measured in deionized water at a 1:4 (*w*/*v*) ratio using a pH meter (FE28, Mettler Toledo, Switzerland). For lactic acid and glucose quantification, UFG samples were extracted under identical conditions (1:4 *w*/*v* in deionized water) and filtered through a 0.22 μm membrane. Lactic acid was analyzed by UPLC (Acquity I-class, Waters, Milford, MA, USA) as previously described [2], and glucose was measured using an SBA-40X biosensor (Institute of Biology, Shandong Academy of Sciences, Jinan, China). For acetic, butyric, and caproic acid analysis, UFG samples were mixed with 60% ethanol at a 1:4 (*w*/*v*) ratio, sealed in containers, and soaked for 3 h. The extracts were then vortexed, filtered through a 0.22 μm membrane, and analyzed by gas chromatography using an absolute quantitative method with 2-ethylbutyric acid as the internal standard [42]. In addition, the four major esters derived from the aforementioned acids (ethyl lactate, ethyl acetate, ethyl butyrate, and ethyl caproate) were quantified, as their formation influences the net yields of the precursor acids [43]. The same quantitative method was used for the esters with one critical modification: the extraction solvent was changed from 60% ethanol to 30% methanol to prevent potential extraneous esterification during extraction that could artificially alter the ester profiles. This modification was informed by a previous study, in which 15% methanol was used to extract volatile components from solid pit mud [2]; in the present study, 30% methanol was adopted to account for the high moisture content of UFG. Moreover, methanol has been reported to exhibit high elution efficiency for ester compounds in Baijiu [44]. Spike-recovery tests performed in the liquid extraction system showed that the four esters were recovered at 71.2–105.3% (*n* = 3 per level; relative standard deviation (RSD) of all determinations ≤ 15%), with the lower recoveries observed at the low spike level. Matrix-matched spike-recovery in the solid UFG matrix was not feasible, because spiked standards cannot be uniformly dispersed in the heterogeneous grain matrix and the high native ester background precludes accurate quantification of the spike.

### 2.4. Real-Time Quantitative PCR

The absolute copy number of the 16S rRNA gene in the aged-pit UFG samples were adopted directly from the parallel investigation [36], while those of the new-pit UFG samples were determined in this study using the same protocol as in [36], on a PikoReal real-time PCR system (Thermo Scientific, Waltham, MA, USA). Accordingly, the absolute abundance of each genus was calculated by multiplying its relative abundance (from 16S rRNA gene amplicon sequencing) by the qPCR-quantified total 16S rRNA gene copy number of the corresponding sample, and the resulting values were log_10_-transformed for downstream analyses. The high length heterogeneity and copy-number variability of the ITS region among fungal taxa preclude reliable inter-taxonomic comparison and absolute quantification [44,45]. Alternative calibration approaches, such as microscopic cell counting and fungal-specific single-copy markers, were also considered; however, they are impractical for the solid and heterogeneous UFG matrix, in which fungal cells occur predominantly as mycelia embedded within the grain substrate, and single-copy markers lack sufficient sensitivity and coverage for the diverse, largely uncultured fungal assemblages in pit fermentations. Therefore, fungal community composition was characterized by ITS amplicon sequencing alone, without qPCR-based absolute quantification. To mitigate the resulting compositional bias, all fungal analyses were based on relative abundances interpreted through rank-based (Spearman) statistics rather than absolute values (Section 2.5).

### 2.5. Statistical Analysis

Redundancy analysis (RDA) was performed in Canoco 5.0 to identify the key physicochemical drivers of (i) prokaryotic community succession and (ii) the accumulation of butyric and caproic acids in UFG. For each objective, a separate RDA model was constructed: in the first model, genus-level prokaryotic community composition served as the response matrix and all physicochemical parameters as explanatory variables; in the second model, the concentrations of butyric and caproic acids constituted the response matrix, with the physicochemical parameters excluding these two acids as explanatory variables. To account for the phasic dynamics of UFG fermentation, both models were run separately for the three fermentation stages (early: 2–8 d; middle: 10–20 d; late: 30–60 d), with the stages defined according to the phasic community succession observed in this study (Section 3.4). The significance of each model and of individual explanatory variables was assessed by Monte Carlo permutation tests (999 permutations), with *p* < 0.05 considered significant.

Unless otherwise stated, between-group differences at each time point were assessed by one-way ANOVA, after verification of normality and homogeneity of variance, with pit as the experimental unit (*n* = 3 per group), and *p*-values were adjusted by Tukey’s HSD (significance: *p* < 0.05). Pearson and Spearman correlation analyses were performed in R (version 4.3.3) to identify genera associated with caproic acid metabolism and esterification (Files S1 and S2). Pearson correlation was applied to prokaryotes, whose abundances were obtained by qPCR-based absolute quantification, whereas Spearman correlation was applied to eukaryotes, whose abundances were derived from ITS amplicon sequencing as relative abundances. For prokaryotes, correlations were computed between the absolute abundance of each genus and the concentrations of butyric and caproic acids as well as the four major esters (ethyl acetate, ethyl lactate, ethyl butyrate, and ethyl caproate), thereby covering both direct producers and potential synergistic partners. For eukaryotes, correlations were computed between the relative abundance of each genus and the four esters only, given that yeasts are not known to produce caproic acid directly. Correlations were calculated separately within each fermentation stage (early: 2–8 d; middle: 10–20 d; late: 30–60 d) to account for phasic dynamics. Genera whose mean relative abundance (averaged over the three replicate pits) exceeded 0.5% in at least one sample were included in the analysis, with the traditional CPB genus *Caproiciproducens* included regardless of its abundance. Correlations with *p* < 0.05 were considered significant. In addition, to enable a fair comparison between prokaryotic and eukaryotic co-occurrence networks, prokaryotic and eukaryotic networks were inferred separately from genus-level abundance data using the same Spearman’s rank correlation analysis. Only taxa with significant correlations (Spearman’s r > 0.6 or r < −0.6, *p* < 0.05) were retained as network edges, and the resulting networks were visualized using Cytoscape (v3.10.2).

### 2.6. Data Availability

The 16S rRNA and ITS gene amplicon sequencing data for UFG samples from aged and new pits have been deposited in the NCBI Sequence Read Archive (SRA) under BioProject accession numbers PRJNA1363719 (SRS27107350–SRS27107430, with a gap at SRS27107379–SRS27107388) and PRJNA1459030 (SRX33152011~SRX33152082), respectively.

## 3. Results and Discussion

### 3.1. UFG Microbial Community Structure, Diversity, and Caproiciproducens Abundance: Aged vs. New Pits

#### 3.1.1. Dynamics of UFG Prokaryotic Community Structure, α-, and β-Diversity

**Figure 1:**
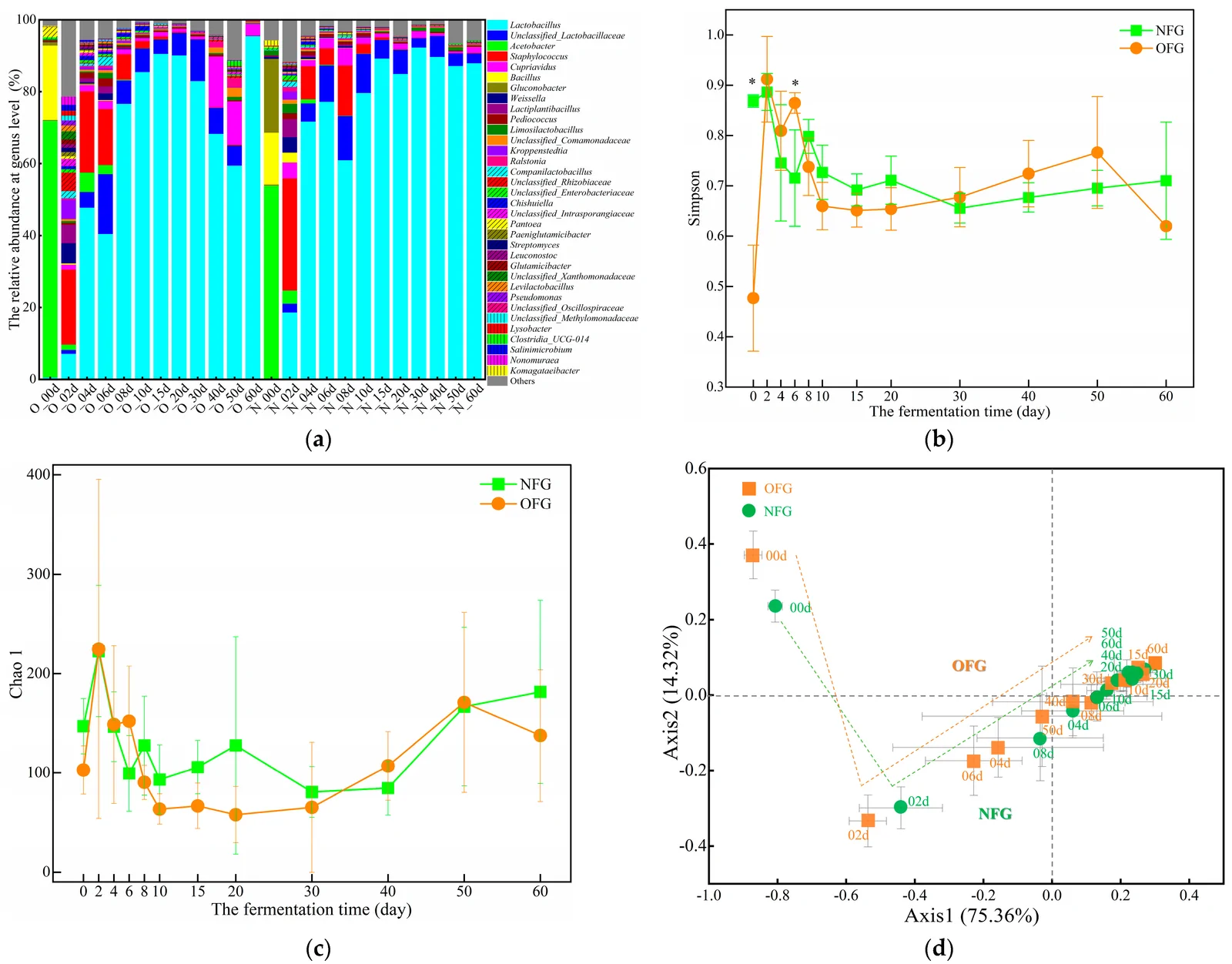
Dynamics of prokaryotic communities, α- and β- diversity in UFG from new (NFG) and aged (OFG) pits during fermentation. (**a**) Genus-level community succession; (**b**) Simpson index; (**c**) Chao1 index (error bars in (**b**,**c**) represent the standard deviation (SD) of three replicate pits, *n* = 3); (**d**) PCA ordination of the genus-level community structures (error bars represent the standard error (SE) of the three replicate pits). Asterisks in (**b**) indicate significant differences between new- and aged-pit samples at the corresponding time points (*p* < 0.05, ANOVA).

Overall, 25,070–133,029 clean 16S rRNA gene sequences per sample were obtained. For α- diversity analysis, all samples were rarefied to 24,000 sequences (Table S3). The sequencing depth was adequate since the Good’s coverage sequences in all samples were above 99%. The UFG microbiota from both new and aged pits followed a consistent directional succession. At day 0, samples from the two pit types were similar, both dominated by the aerobic genera *Acetobacter* (phylum Pseudomonadota, formerly Proteobacteria) and *Bacillus*, indicating that the UFG had just been loaded into the pits and that anaerobic fermentation had not yet commenced; day 0 was thus designated the lag phase. From day 2 onward, Pseudomonadota was rapidly supplanted by Bacillota (formerly Firmicutes), which exceeded 80% by day 4 and stabilized from day 10 onward (Figure S2). This phylum-level transition was mirrored at the family level by a shift from Acetobacteraceae to Lactobacillaceae, and at the genus level by the replacement of *Acetobacter* with *Lactobacillus*, the latter stabilizing after day 10 until the end of fermentation (Figure 1a and Figure S3), consistent with prior findings [4,29,30,32,35]. This shift reflects the transition of the UFG microbiota from aerobic to anaerobic fermentative communities during the onset of active fermentation. More specifically, during this rapid growth phase (days 2–8), Cyanobacteria (aerobic/oxygenic phototrophs), the predominantly aerobic families Staphylococcaceae, Bacillaceae, and Thermoactinomycetaceae, and the facultatively anaerobic genera *Staphylococcus*, *Weissella*, and *Lactiplantibacillus* exhibited transient dominance, while *Lactobacillus* followed a sigmoidal growth curve to achieve absolute dominance (Figure 1a).

During days 10–20, the UFG microbiota remained stable in both pit types, with *Lactobacillus* maintaining absolute dominance and unclassified Lactobacillaceae persisting as the principal strictly or facultatively anaerobic taxa, while low-abundance aerobic or facultatively anaerobic groups (e.g., *Cupriavidus*) persisted throughout the fermentation cycle. After day 30, the communities remained largely stable but exhibited subtle divergence: transient rebounds of Pseudomonadota at the phylum level (Figure S2), significantly elevated Burkholderiaceae at the family level during days 40–50 (Figure S3), and, at the genus level, a rebound of *Cupriavidus* [46]—a member of Burkholderiaceae—in aged-pit UFG (Figure 1a). Based on these phasic dynamics, fermentation was divided into a lag phase (day 0), an early stage (days 2–8), a mid stage (days 10–20), and a late stage (days 30–60), which are used in all subsequent stage-resolved analyses.

Simpson diversity and Chao1 richness remained low during the lag phase, increased rapidly by day 2, then declined gradually while remaining relatively high during the early stage (days 2–8). Both parameters stabilized during the mid stage (days 10–20), increased slightly during the late stage (days 30–60), and peaked at days 50–60, with no significant differences between new and aged pits during middle and late stages (Figure 1b,c). Significant differences between the two pit types were confined to the Simpson index and to two time points: at day 0, Simpson diversity was significantly higher in new-pit UFG, whereas at day 6 it was significantly higher in aged-pit UFG (Figure 1b); Chao1 richness showed no significant between-pit differences at any time point (Figure 1c). This pattern is largely consistent with previous findings on the initial decrease and subsequent recovery of FG microbial diversity [29]. PCA of the prokaryotic community structure (Figure 1d) revealed that the divergence between the two pit types was primarily driven by Axis 1 (75.36%). Both pit types exhibited parallel trajectories: samples shifted from the negative end of Axis 1 (0–2 d) toward the positive end (4–60 d), reflecting a consistent directional succession broadly consistent with Hu et al. [33]. Aged-pit UFG consistently occupied more positive Axis 1 positions than new-pit UFG, with the offset marginally widening during the late stage (days 30–60), indicating minor yet persistent discrepancies in community maturity between the two pit types, in agreement with the compositional dynamics (Figure 1a).

Overall, the prokaryotic community of UFG exhibited phasic dynamics consistent with prior fermentation studies [4,23,33,35,46,47]. Xu et al. and Hu et al. both divided fermentation into two stages at 25 d [4,33]; others further subdivided the early stage, although the boundaries varied with sampling designs (e.g., 0–12 d [47] vs. 0–8 d [48]). Within the same fermentation framework, 0 d (prior to the onset of fermentation) was designated as the lag phase, with subsequent fermentation divided into early (2–8 d), middle (10–20 d), and late (30–60 d) stages.

#### 3.1.2. Abundance of *Caproiciproducens* (Classical CPB): Aged vs. New Pits

**Figure 2:**
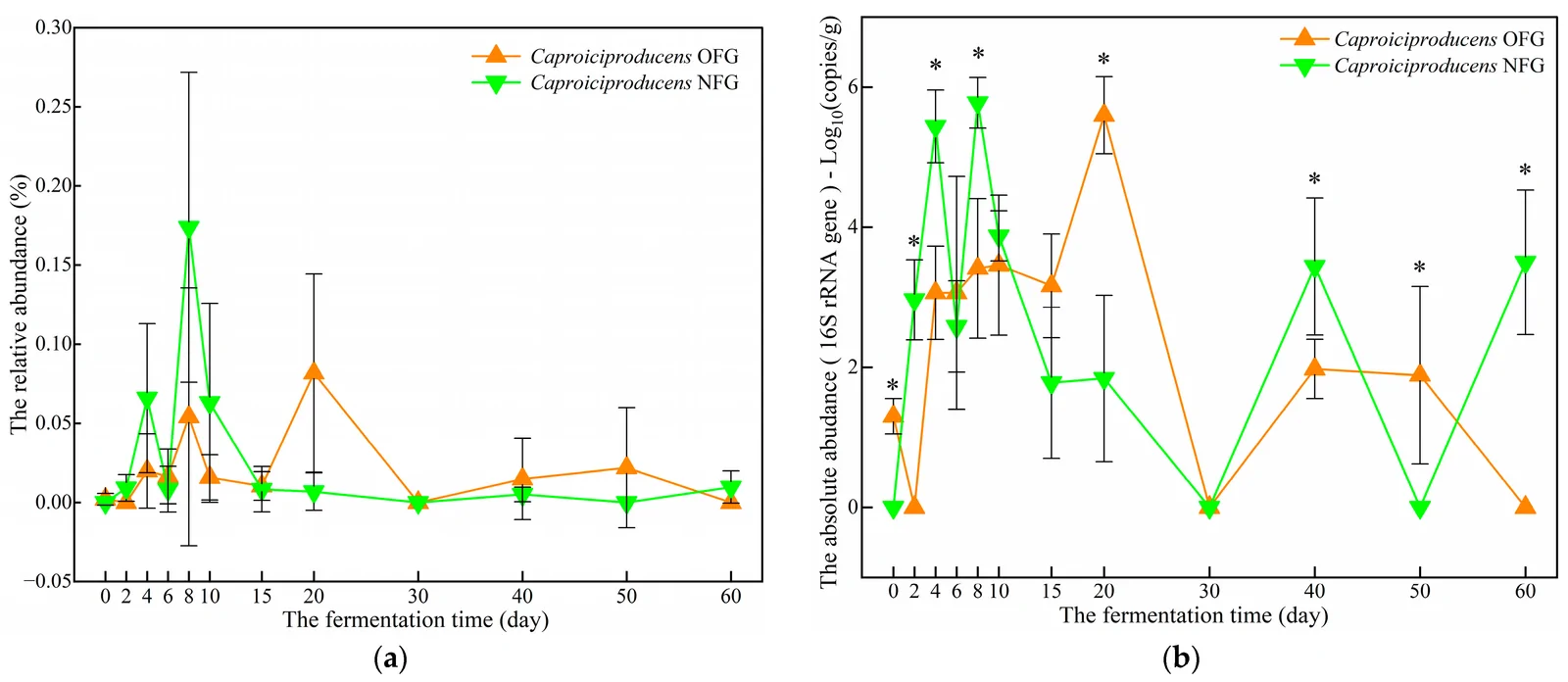
Relative and absolute abundances of *Caproiciproducens* (traditional CPBs) in UFG from new and aged pits during fermentation. (**a**) Relative abundance; (**b**) absolute abundance. Data are presented as means ± SD (*n* = 3 independent pits per group; error bars represent the standard deviation calculated across the three pits). Asterisks indicate significant differences between new- and aged-pit samples at the corresponding time points (*p* < 0.05, ANOVA).

The traditional CPB genus *Caproiciproducens* was not dominant in UFG from either pit type (Figure 1a); its relative and qPCR-based absolute abundances were therefore examined specifically (Table S7). *Caproiciproducens* remained at low relative abundance throughout fermentation in both aged (0–0.14%) and new (0–0.28%) pits, with no significant difference between them (Figure 2a). Its absolute abundance was also at trace levels (0–6.01 log_10_ copies/g) in both aged and new pits, with zero values indicating that the genus was below the detection limit in those samples, and was largely comparable between the two pit types except at a few time points where aged-pit UFG was significantly higher (Figure 2b). These findings corroborate prior metagenomic evidence that CPBs such as *Caproicibacterium lactatifermentans* are significantly less abundant in upper than in lower FG [29], and are consistent with analogous UFG studies that likewise failed to identify *Caproiciproducens* or related taxa as dominant members [30,31]. Collectively, these results demonstrate that the traditionally recognized CPB genus *Caproiciproducens* occurs at very low abundance in UFG. This is a critical observation, because substantial caproic acid accumulation was nonetheless detected in the UFG (Section 3.3.1), implying that microorganisms other than the traditional CPB genus drive caproic acid synthesis in this layer—an inference that is examined below by integrating PICRUSt2-based functional prediction with physicochemical analyses.

#### 3.1.3. UFG Eukaryotic Community Structure and Diversity: Aged vs. New Pits

Since eukaryotic communities are the primary agents of esterification for the four major esters [24,49,50], they were analyzed as potential drivers of esterification affecting both caproic acid synthesis substrates and products. Fungal dynamics were examined across phylum, family, and genus levels (Table S4). At all three levels, communities from both pit types followed a consistent directional succession, yet exhibited distinct taxonomic signatures. At the phylum level, Ascomycota overwhelmingly dominated throughout CSFB fermentation, with an average relative abundance of 88.57% in aged-pit UFG versus 80.10% in new-pit UFG. Unclassified_Fungi and Mucoromycota remained minor yet persistent constituents (Figure S4).

**Figure 3:**
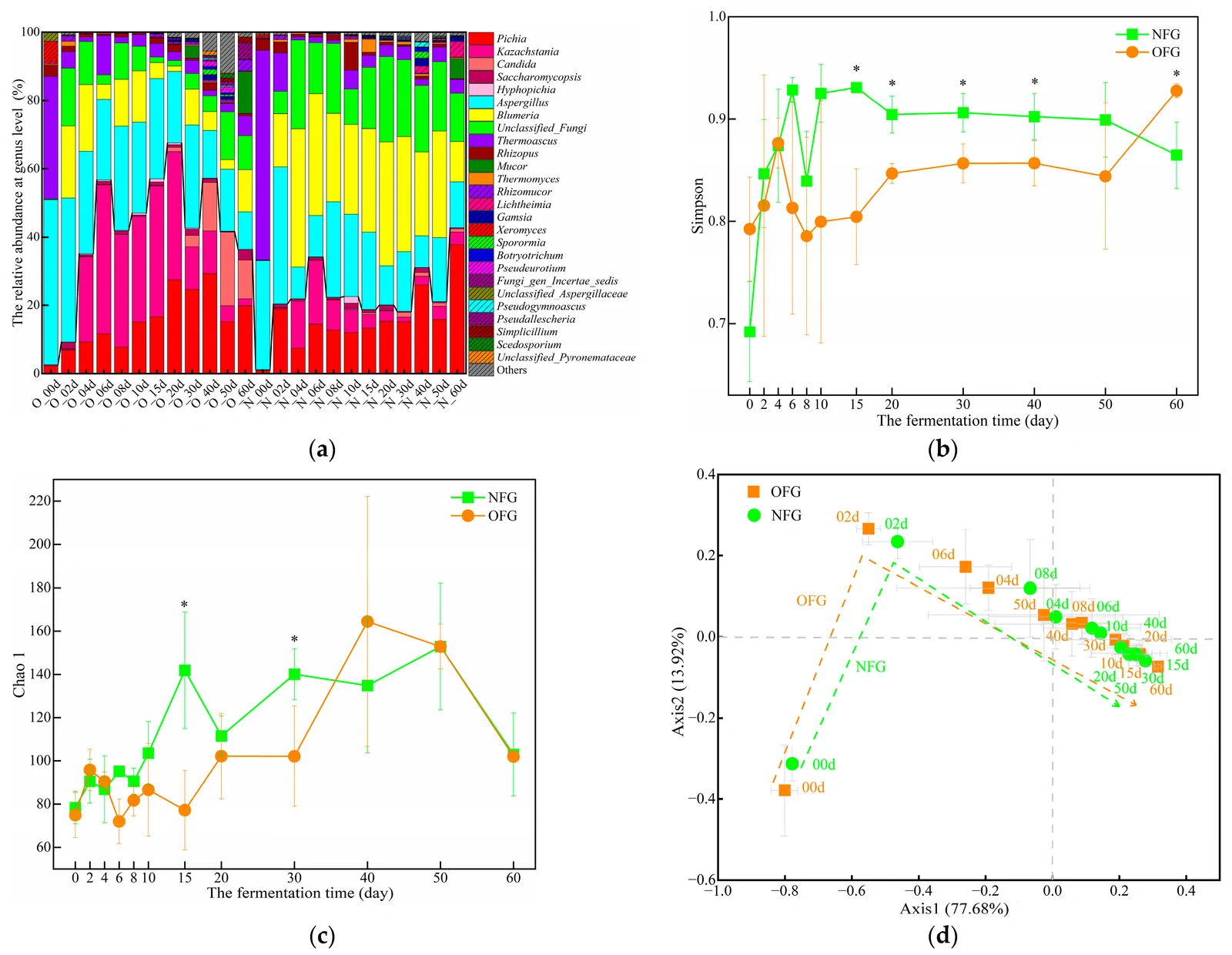
Dynamics of eukaryotic communities and alpha diversity in UFG from new and aged pits during fermentation. (**a**) Genus-level community succession; (**b**) Simpson index; (**c**) Chao1 index; (**d**) PCA ordination of genus-level eukaryotic community structures in UFG from new and aged pits during fermentation. Data in (**b**,**c**) are presented as means ± SD (*n* = 3 independent pits per group); data in (**d**) are presented as means ± SE (*n* = 3 independent pits per group). Asterisks in (**b**,**c**) indicate significant differences between new- and aged-pit samples at the corresponding time points (*p* < 0.05, ANOVA).

At 0 d (lag phase), *Aspergillus* and *Thermoascus* overwhelmingly dominated UFG samples (Figure S5 and Figure 3a), a composition closely resembling the eukaryotic community of Daqu [51], suggesting that the initial eukaryotic microbiota primarily originated from Daqu. Subsequently, *Aspergillus*, which is associated with starch saccharification [52], maintained stable relative abundance throughout fermentation; notably, its average abundance was higher in aged-pit UFG (27.07% vs. 19.89%), suggesting a competitive advantage of *Aspergillus* in the mature pit environment. Unlike *Aspergillus*, the non-Saccharomyces yeasts *Pichia* and *Candida* exhibited a sigmoidal growth curve over a more extended log phase than the bacterial genus Lactobacillus (Figure 1a). Specifically, their relative abundance gradually increased during the early to middle stages (days 2–30) and subsequently declined during the late stage (days 30–60). Aged-pit UFG harbored higher relative abundances of these yeasts than new-pit UFG; conversely, non-fermentation-beneficial taxa *Blumeria* (25.99% in new vs. 9.29% in aged pits) and *unclassified fungi* (16.49% in new vs. 7.23% in aged pits) remained consistently more abundant in new-pit UFG throughout the entire fermentation cycle (Figure 3a). Given that identical Daqu was used in both pits, the low abundance of these yeasts in new-pit UFG likely reflects input from the fungal community of the immature wall pit mud adjacent to the UFG. Indeed, upper-layer wall pit mud fungal composition differs markedly from bottom pit mud [53], and wall pit mud in both pit types supports higher fungal abundance and diversity than bottom pit mud or lower-layer wall pit mud [3,54]. Taken together, the eukaryotic succession can be divided into three phases: a lag phase (day 0) dominated by Daqu-derived *Aspergillus* and *Thermoascus*; a prolonged growth phase (days 2–30) characterized by the progressive enrichment of non-Saccharomyces yeasts; and a late phase (days 30–60) marked by yeast decline and community stabilization. Unlike the prokaryotic succession, which comprised four distinct phases with a clear stationary stage (days 10–20; Section 3.1.1), the eukaryotic community exhibited no discrete stationary phase: its growth phase extended across both the early and middle prokaryotic stages, and its late phase coincided with the prokaryotic late stage. Notably, these fungal phases therefore lag behind the prokaryotic succession—most visibly in the delayed peak of yeasts relative to *Lactobacillus* (Figure 1a)—consistent with the slower growth of eukaryotes under the same fermentation conditions.

Regarding eukaryotic richness and diversity, the less mature new-pit UFG harbored a more diverse fungal community during the early to middle stages of fermentation, as evidenced by significantly higher Simpson diversity indices than aged-pit UFG (Figure 3b). Correspondingly, new-pit UFG exhibited higher eukaryotic Chao1 richness during the middle stage (Figure 3c). By the end of fermentation, however, both Simpson and Chao1 indices converged between new and aged-pit UFG, with no significant difference detected. PCA further revealed that, although the UFG eukaryotic communities of new and aged pits exhibited distinct successional dynamics, both followed clear trajectories along Axis 1 (77.68%), converging toward late fermentation while maintaining distinct clustering (Figure 3d).

In summary, the eukaryotic community of UFG exhibited a phasic succession that mirrored—but temporally lagged—the prokaryotic dynamics: the amylolytic genus *Aspergillus* remained relatively stable throughout fermentation, whereas non-Saccharomyces yeasts as a whole (*Pichia*, *Wickerhamomyces*, *Saccharomycopsis*, *Kazachstania*, and *Candida*) followed a sigmoidal curve with a more extended logarithmic phase than *Lactobacillus*. Both *Aspergillus* and non-Saccharomyces yeasts, known for their robust ester-producing capacities, were relatively more abundant in aged-pit UFG, suggesting their potential contribution to esterification; this hypothesis will be subsequently validated through physicochemical parameter analysis.

### 3.2. PICRUSt2-Predicted Fatty Acid Biosynthesis and Ester Formation: Aged vs. New Pits

#### 3.2.1. PICRUSt2-Predicted Fatty Acid Biosynthesis: Aged vs. New Pits

**Figure 4:**
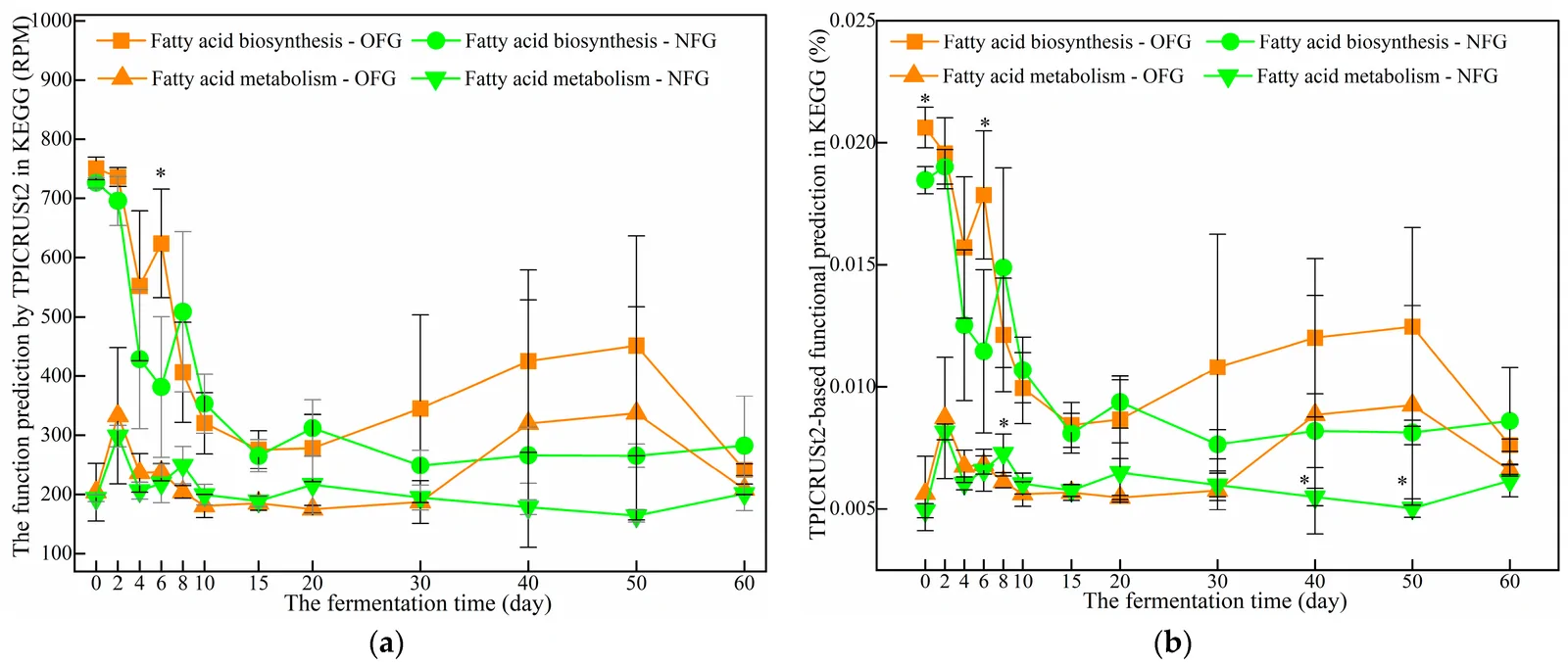
PICRUSt2-predicted dynamics of fatty acid biosynthesis and metabolism in UFG from new and aged pits during CSFB fermentation. (**a**) Absolute abundance (reads per million, RPM); (**b**) relative abundance (%). Data are presented as means ± SD (*n* = 3 independent pits per group; error bars represent the standard deviation calculated across the three pits). Asterisks indicate significant differences between the two lines immediately below each asterisk at the corresponding time points (*p* < 0.05, ANOVA).

Caproic acid synthesis in microorganisms is known to proceed primarily via the fatty acid biosynthesis (FAB) or reverse β-oxidation (RBO) pathway [5], which in the KEGG database are represented by the “fatty acid biosynthesis” (ko00061) and “fatty acid degradation” (ko00071) pathways, respectively. PICRUSt2 profiling of the UFG prokaryotic communities based on KEGG Orthology predicted the “fatty acid biosynthesis” pathway but not “fatty acid degradation” (Figure 4; Table S5). Because PICRUSt2 reports pathway abundances at the most specific level without aggregating them into higher-level categories [38], the abundance of “fatty acid biosynthesis” appeared higher than that of its parent category “fatty acid metabolism”. These predictions thus suggest that the fatty-acid-related functional potential in UFG is dominated by the FAB rather than the RBO route, consistent with Tong et al. [3].

Both pit types exhibited similar trajectories of predicted FAB abundance: it peaked during the early stage (days 2–8), declined thereafter, stabilized during the mid stage (days 10–20), and rebounded during the late stage (days 30–50) (Figure 4), suggesting potential caproic acid synthesis activity in both the early and late fermentation periods. The only significant between-pit difference occurred at day 6, when the predicted FAB abundance in aged-pit UFG was significantly higher than that in new-pit UFG; at all other time points the two pit types were statistically comparable, although aged-pit UFG showed marginally higher mean values. This FAB-dominated prediction contrasts with the prevailing view that traditional CPBs synthesize caproic acid via the RBO pathway [7,8,9,55]. Together with the trace abundance of *Caproiciproducens* in UFG (Section 3.1.2), this discrepancy suggests that taxa other than the traditionally recognized CPB genus may contribute to caproic acid synthesis in the UFG.

In addition, Pearson correlations between the predicted FAB abundance and the absolute abundances of dominant prokaryotic genera revealed that positive correlations predominated in the early stage, followed by the late stage (Figure S6), mirroring the phasic dynamics of the predicted FAB abundance (Figure 4). The positively correlated genera included numerous lactic acid bacteria (LAB)—*Lactobacillus*, *Weissella*, *Lactiplantibacillus*, and *Limosilactobacillus*—as well as previously unconfirmed taxa such as *Acetobacter*, *Bacillus*, and *Gluconobacter*, which are hypothesized to may directly or synergistically participate in caproic acid synthesis. For instance, Zhang et al. reported that *Sporolactobacillus* sp. LBM15018 promoted caproic acid production by *Caproicibacterium lactatifermentans* through syntrophic interactions [56]. These predictions remain to be validated by functional approaches, such as metagenomic sequencing or isolate-based assays.

#### 3.2.2. PICRUSt2-Predicted Ester Formation: Aged vs. New Pits

**Figure 5:**
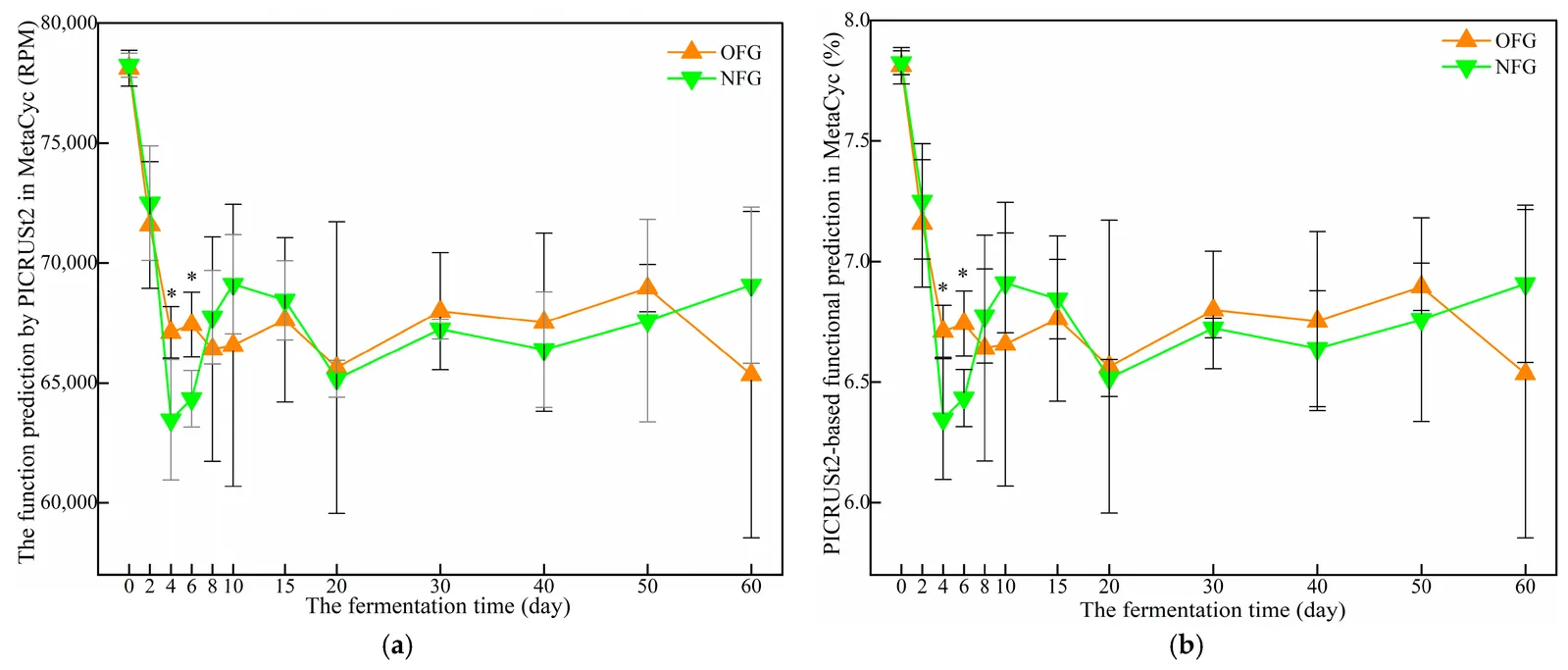
PICRUSt2-predicted dynamics of ester formation in UFG from new and aged pits during CSFB fermentation. (**a**) Absolute abundance (reads per million, RPM); (**b**) relative abundance (%). Data are presented as means ± SD (*n* = 3 independent pits per group; error bars represent the standard deviation calculated across the three pits). Asterisks indicate significant differences between new- and aged-pit samples at the corresponding time points (*p* < 0.05, ANOVA).

PICRUSt2 was used to predict functional profiles from ITS amplicon sequencing data against the MetaCyc database, and the temporal dynamics of predicted esterification enzyme gene abundances in eukaryotic communities were tracked (Figure 5; Table S6), based on esterification enzymes compiled by Yi et al. [24]. Dynamics of predicted ester-formation enzymes followed similar trajectories in both new- and aged-pit UFG, declining during the initial adaptation and early stages (0–8 d) before stabilizing from the mid stage through late stage. Significant divergence was restricted to days 4 and 6, when aged-pit UFG exhibited higher relative abundance than new-pit UFG, likely reflecting the markedly steeper decline in the predicted values in new-pit UFG. Additionally, although mean predicted esterification enzyme gene abundances were marginally higher in new-pit UFG during days 10–20 and in aged-pit UFG during days 20–50, neither showed a significant difference between the two. Whether these predicted functions correspond to actual esterification performance remains to be validated through experimentally determined esterification product analysis, as detailed in the following section.

### 3.3. Physicochemical Properties in UFG: Aged vs. New Pits

#### 3.3.1. Physicochemical Properties Related to Fatty Acid Biosynthesis: Aged vs. New Pits

**Figure 6:**
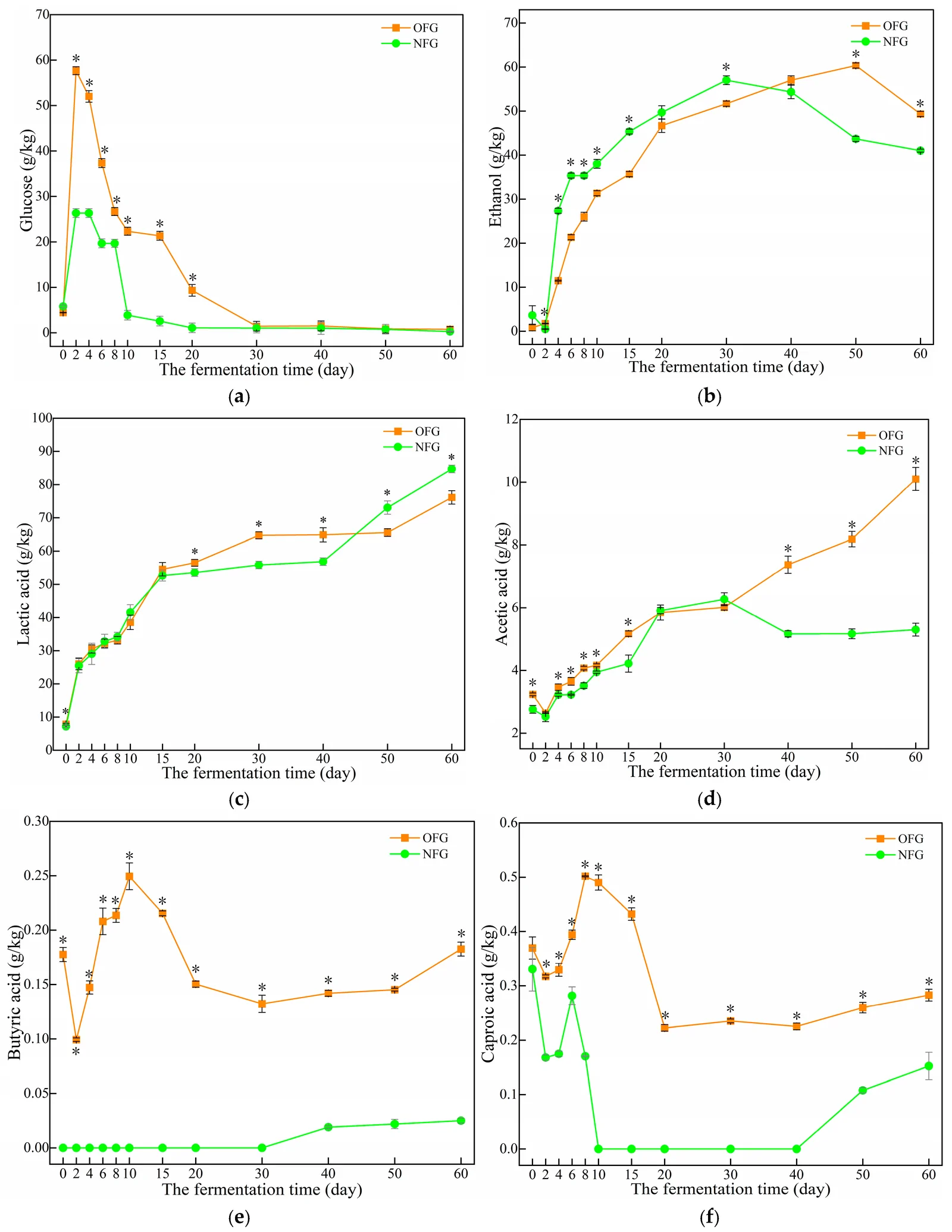
Dynamics of physicochemical properties related to fatty acid biosynthesis in UFG from new and aged pits during CSFB fermentation. (**a**) Glucose; (**b**) ethanol; (**c**) lactic acid; (**d**) acetic acid; (**e**) butyric acid; and (**f**) caproic acid. Data are presented as means ± SD (*n* = 3 independent pits per group; error bars represent the standard deviation calculated across the three pits). Asterisks indicate significant differences between new- and aged-pit samples at the corresponding time points (*p* < 0.05, ANOVA).

At the onset of fermentation (day 0), no significant differences were detected in temperature, starch, glucose, or ethanol contents between aged- and new-pit UFG (Figure 6a,b and Figure S7a,b; Table S1), indicating consistent feedstock processing prior to fermentation. By contrast, aged-pit UFG displayed significantly higher initial concentrations of NH_4_^+^-N, lactic acid, acetic acid, and butyric acid, whereas new-pit UFG exhibited a significantly higher initial pH (Figure 6c–e and Figure S7c,e). These discrepancies reflect the cumulative metabolic residues of sequential batch fermentation and repeated FG recycling across pit ages, which collectively account for the lower initial pH of aged-pit UFG.

During early stage (2–8 d), starch contents in UFG of both pit types underwent a rapid initial decline (Figure S7b), presumably driven by amylolytic fungi such as *Aspergillus* and *Rhizomucor*, both of which maintained stable populations throughout this phase (Figure 3a). The glucose liberated from starch hydrolysis—a direct carbon substrate for caproic acid metabolism—peaked shortly after day 2 and subsequently decreased rapidly (Figure 6a). Notably, aged-pit UFG retained significantly higher residual glucose than new-pit UFG during this period. Taken together with the moisture dynamics (Figure S7d), this pattern suggests that new-pit UFG experienced more rapid starch saccharification accompanied by enhanced water release from the solid matrix [20], resulting in a significantly higher moisture content than in aged-pit UFG. The combination of elevated moisture and accelerated saccharification likely intensified microbial metabolic activity in new-pit UFG, leading to rapid glucose consumption and consequently lower residual glucose concentrations (Figure 6a). Concurrently, rapid metabolic heat release drove a sharp temperature rise (Figure S7a), while ethanol, lactic acid, and acetic acid accumulated rapidly (Figure 6b–d), reflecting the log growth of ethanol-producing yeasts (e.g., *Pichia* and *Kazachstania*) and LAB (primarily *Lactobacillus*) (Figure 1a and Figure 3a). Butyric and caproic acids also increased sharply during this phase (Figure 6e,f), with aged-pit UFG exhibiting significantly higher concentrations than new-pit UFG, indicating active caproic acid synthesis that was more robust in the aged-pit system. However, the traditional CPB genus *Caproiciproducens* showed only a marginal, non-significant increase in relative abundance during this stage, with no significant difference between aged- and new-pit UFG (Figure 2). This discrepancy between *Caproiciproducens* abundance and caproic acid production suggests that the enhanced caproic acid synthesis in aged-pit UFG may derive from more vigorous synergistic metabolism among other CPBs or associated microbiota rather than from *Caproiciproducens* alone [5,57,58]—consistent with the early-stage peak of predicted fatty acid biosynthesis potential (Section 3.2.1). Meanwhile, both NH_4_^+^-N and pH rose rapidly (Figure S7c,e). The increase in NH_4_^+^-N presumably reflects active microbial protein degradation during this phase [59], which would be expected to elevate pH [60]; nevertheless, the more vigorous caproic acid synthesis in aged-pit UFG counteracted this alkalizing effect, yielding a slightly lower pH than in new-pit UFG. Finally, a strain isolated from UFG in our laboratory, *Ligilactobacillus acidipiscis* YHS1A (16S rRNA gene accession No. PQ626723.1), has been shown to produce short-chain fatty acids [36]. In addition, other as-yet-unknown caproic acid-producing strains remain to be investigated.

**Figure 7:**
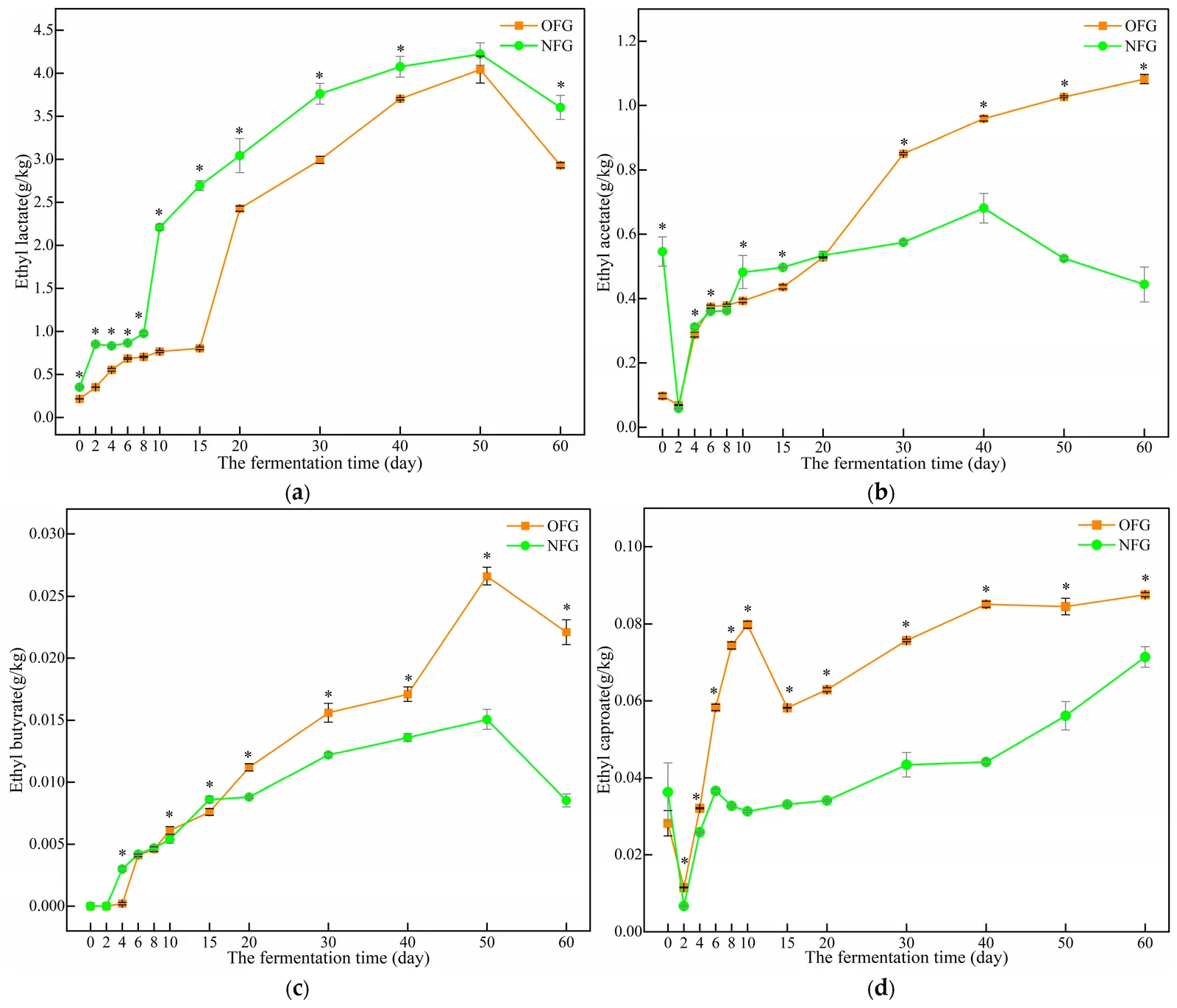
Dynamics of the four major esters in UFG from new- and aged-pits during CSFB fermentation. (**a**) Ethyl lactate; (**b**) ethyl acetate; (**c**) ethyl butyrate; and (**d**) ethyl caproate. Data are presented as means ± SD (*n* = 3 independent pits per group; error bars represent the standard deviation calculated across the three pits). Asterisks indicate significant differences between new- and aged-pit samples at the corresponding time points (*p* < 0.05, ANOVA).

During the mid stage, temperatures in both pit types stabilized at consistently high levels (31.20–32.8 °C and 31.30–32.8 °C in new- and aged-pit UFG, respectively), with no significant difference between pit types (Figure S7a). Approaching day 30, ethanol continued to accumulate and gradually approached a plateau, with new-pit UFG exhibiting a faster rise (Figure 7b). This pattern presumably reflects the plateauing of ethanol-producing yeasts such as *Pichia* and *Kazachstania* during this phase (Figure 3a), suggesting more vigorous yeast metabolism in new-pit UFG. Concurrently, lactic and acetic acids continued to accumulate, transiently reaching a plateau (Figure 6c,d); notably, lactic acid rose more rapidly in aged-pit UFG, presumably because LAB populations peaked during this period with higher metabolic activity in aged-pit UFG (Figure 1a). Comparative analysis of *Lactobacillus* abundance between the two pit types (Figure S8) revealed no significant difference, suggesting that the key distinction lies not in population size but rather in microbial adaptability or community-level synergistic interactions. Meanwhile, starch continued to decline at a reduced rate compared with the early stage, gradually approaching minimum values, with new-pit UFG showing faster depletion (Figure S7b). This decelerated yet ongoing saccharification likely reflects the antimicrobial effects of accumulated ethanol and lactic acid, together with the progressively intensified anaerobic environment, which collectively suppress the saccharifying activity of aerobic or facultatively aerobic amylolytic fungi. Concomitantly, additional water released from starch hydrolysis enabled new-pit UFG to reach peak moisture earlier than aged-pit UFG (Figure S7d), while glucose was gradually depleted during this period (Figure 6a). The concurrent depletion of starch and glucose substrates alongside the plateauing of ethanol, lactic acid, and acetic acid production during this phase is consistent with previously reported fermentation dynamics [6,31,33,35,61]. Notably, butyric and caproic acid synthesis declined sharply during this stage (Figure 6e,f), suggesting that elevated lactic acid and ethanol concentrations create a metabolic environment unfavorable for caproic acid synthesis. Finally, NH_4_^+^-N in both pit types continued to increase toward a transient plateau, but at a markedly reduced growth rate relative to the early stage, with aged-pit UFG maintaining significantly higher concentrations (Figure S7c). Meanwhile, the progressive accumulation of lactic and acetic acids during this phase drove a rapid pH decline (Figure S7e), which outweighed the alkalizing effect of NH_4_^+^-N.

During late stage (30–60 d), starch and glucose in both aged- and new-pit UFG were depleted to minimum levels (Figure S7b and Figure 6a). Ethanol exhibited a gradual decline after peaking (Figure 6b). However, lactic acid displayed a secondary resurgence in both pit types, presumably attributable to the ethanol tolerance of LAB (such as *Lactobacillus*) [62], which enabled continued acid production after alcoholic fermentation had subsided. This sustained lactic acid synthesis during the late stage of FG fermentation was similarly observed in previous studies [4,30,34]. Although moisture in new-pit UFG increased earlier than in aged-pit UFG, the peak values did not differ significantly between the two (Figure S7d). Notably, aged-pit UFG showed significantly higher acetic acid accumulation than new-pit UFG during this stage (Figure 6d). This may reflect differences in acetic acid-producing bacterial abundance [63] or, assuming comparable *Lactobacillus* levels, divergent metabolic modes or species compositions—heterolactic fermentation in aged-pit UFG versus homolactic fermentation in new-pit UFG [64]. The opposing trends in acetic and lactic acid dynamics during late fermentation (Figure 6c,d) support this hypothesis, but further validation is needed. Thus, late-stage UFG (30–60 d) harbored sufficient carbon sources and electron donors (lactic acid, ethanol, and glucose) along with the electron acceptor acetic acid, enabling trace accumulation of butyric and caproic acids (Figure 6e,f).

This late-stage accumulation aligns with the established view that caproic acid synthesis occurs predominantly during late fermentation [18,19,20,31,33], and, together with the early-stage accumulation described above, corroborates the biphasic synthesis pattern inferred from the PICRUSt2 predictions (Section 3.2.1). The relatively low caproic acid yield likely reflects the low abundance of the traditional CPB genus *Caproiciproducens* in UFG [29,32], whereas the role of non-traditional CPBs remains to be investigated. This late-stage acid production presumably derived from continued metabolism of residual starch and glucose following the cessation of ethanol fermentation. Concurrently, NH_4_^+^-N accumulation driven by grain protein degradation peaked at day 40 in new-pit UFG and day 50 in aged-pit UFG, respectively, with the final concentration being significantly higher in the latter (Figure S7c). This is presumably associated with a higher abundance of amino acid-degrading bacteria in aged-pit UFG, such as *Staphylococcus* and *Pseudomonas* (Figure 1a), which possess deaminating capabilities (converting amino acids to NH_4_^+^-N) [65,66]. Finally, pH exhibited a slight secondary rise during days of 50–60 (Figure S7e), yet aged-pit UFG maintained consistently and significantly higher values than new-pit UFG throughout this period. Given that caproic acid production utilizing lactic acid as a carbon source has been reported to elevate pH [5,11], the observed pH rise is presumably linked to caproic acid synthesis by putative CPBs using lactate as the carbon source following glucose depletion (Figure 6e,f). This inference requires verification in future studies.

#### 3.3.2. Physicochemical Properties Related to Ester Formation: Aged vs. New Pits

The substrates (lactic acid and acetic acid), intermediate (butyric acid), and end product (caproic acid) involved in caproic acid synthesis can each undergo esterification to form the corresponding esters (ethyl lactate, ethyl acetate, ethyl butyrate, and ethyl caproate). Concurrently, the sequestration of these acids into ester forms may modulate caproic acid synthesis by altering substrate and product availability. This section accordingly focuses on the dynamics of these four esters (Figure 7). First, ethyl lactate, the most abundant of the four esters, accumulated slowly during the early stage before entering a logarithmic esterification phase in the mid stage, with continuous accumulation peaking at day 50 during late fermentation. Relative to its precursor lactic acid, the esterification rate was low (1.97–5.19% and 1.02–4.49% in new- and aged-pit UFG, respectively). The dynamics of ethyl lactate paralleled the proliferation of lactic acid-producing bacteria—particularly *Lactobacillus* [67]—consistent with reports that *Lactobacillus* promotes ester formation [23,48]. However, because *Lactobacillus* abundance did not differ significantly between the two pit types (Figure S8), the consistently higher ethyl lactate levels in new-pit UFG throughout fermentation (Figure 7a)—despite comparable lactic acid concentrations—suggest that factors beyond precursor availability, such as differential esterification activity, may contribute to ethyl lactate accumulation; however, in the absence of enzyme activity measurements, this interpretation remains speculative.

Similarly, ethyl acetate exhibited comparable dynamics (esterification rates: 1.55–11.90% in new-pit vs. 1.75–8.79% in aged-pit UFG) (Figure 7b). Given the strong correlation between acetic acid synthesis and *Lactobacillus* [67], the higher ethyl acetate levels in aged-pit UFG during the mid to late fermentation mirrored differential acetic acid production (Figure 6d), consistent with a coupling of ethyl acetate formation to acetic acid availability. In contrast, ethyl butyrate and ethyl caproate occurred at relatively low concentrations, although a large proportion of their precursor acids was sequestered as esters: the esterification rates of ethyl butyrate and ethyl caproate fluctuated widely in new-pit UFG (0–100.00% and 3.13–100.00%, respectively) but varied within much narrower ranges in aged-pit UFG (0–12.22% and 2.84–23.33%, respectively), with final levels significantly higher in aged-pit UFG. This disparity presumably reflects differential synthesis and accumulation of their precursors, butyric and caproic acids, between the two pit types (Figure 6e,f). Specifically, ethyl caproate dynamics tracked caproic acid during early fermentation (2–8 d) but subsequently diverged, maintaining a steady increase through mid to late fermentation (Figure 7d). We hypothesize that early phase accumulation was primarily driven by caproic acid availability, whereas sustained production during later stages resulted from synergistic effects of a stabilized microbial community (including esterification-related genera such as *Lactobacillus*, *Pichia*, *Kazachstania*, and *Candida* [48,49,50]), and possibly enhanced esterase activity, and elevated ethanol concentrations (although direct evidence for elevated enzyme activity is currently lacking). The synthesis pattern of ethyl butyrate diverged markedly from that of butyric acid (Figure 6e): whereas butyric acid peaked in aged-pit UFG at day 10 before declining to low stable levels and remained at trace levels in new-pit UFG, ethyl butyrate accumulated progressively in both pit types (Figure 7c). We hypothesize that the inherently low butyric acid production in new-pit UFG—attributable to an underdeveloped chain-elongation community—created substrate competition between two pathways: chain elongation to caproic acid and esterification to ethyl butyrate. Because esterification is catalyzed by highly active alcohol acyltransferases (AAT) via a shorter metabolic route than the chain-elongation cycle, the limited butyric acid pool may be preferentially channeled toward esterification. It should be emphasized, however, that near-zero free butyric acid concentrations need not indicate an absence of synthesis: they may equally reflect rapid turnover, in which production is immediately balanced by consumption (esterification), or partial contribution from chemical (non-enzymatic) esterification between butyric acid and the high ethanol concentrations in UFG. Moreover, because measured ester concentrations represent net accumulation—synthesis minus hydrolysis, microbial consumption, and potential volatilization—at discrete sampling points of a spatially heterogeneous matrix, the present data alone cannot establish the direction or relative rates of the underlying metabolic fluxes. Distinguishing among these mechanisms will require enzyme activity assays and isotope-labeling experiments (e.g., ^13^C-butyric acid tracing), which we identify as priorities for future work. Consequently, ethyl butyrate accumulated to levels comparable to those in aged-pit UFG through day 40, even as butyric acid remained negligible and caproic acid synthesis was suppressed. Furthermore, the acid-to-ester ratio may influence aromatic ester release through intermolecular interactions [68], a mechanism requiring further investigation. Regarding ester quantification, although spike-recovery tests in the liquid extraction system confirmed quantitative recovery of the four esters, and internal-standard correction was applied prior to injection, matrix-matched recovery in the solid UFG matrix could not be assessed owing to the technical constraints described in Section 2.3. It should therefore be emphasized that the ester concentrations reported here are best interpreted as quantitative values within a consistent analytical framework: because the identical extraction procedure was applied uniformly to all samples, any residual extraction bias would affect both pit groups equally and would not compromise the relative comparisons (new vs. aged pits) on which our conclusions are based. The observed ester dynamics are, moreover, consistent with the microbial succession patterns observed in this study and with the ester profiles reported in previous studies on CSFB fermentation [4,42].

In summary, the four esters differed significantly in both content and synthesis dynamics between aged- and new-pit UFG, with three of the four esters attaining higher final levels in aged-pit UFG, whereas ethyl lactate was consistently higher in new-pit UFG (Figure 7), consistent with previous findings [4]. Notably, although ethyl butyrate also attained a significantly higher final content in aged-pit UFG, it exhibited a markedly divergent pattern from its precursor butyric acid, particularly in new-pit UFG where butyric acid was nearly undetectable yet ethyl butyrate continued to accumulate, indicating a distinct metabolic decoupling that merits further study. Finally, because esterification sequesters part of the synthesized acids, the summed molar quantities (free acids plus their ethyl esters) provide a more comprehensive representation of actual microbial synthesis than free acid concentrations alone (Figure S9); accordingly, these combined values were used in the subsequent correlation and RDAs. Overall, this section provides the dynamics of the four major ethyl esters in UFG during fermentation—previous studies having relied predominantly on non-targeted, semi-quantitative profiling [29,30]—with the caveat that the absolute values are best regarded as semi-quantitative estimates and that interpretive emphasis is placed on the observed patterns and relative differences between pit groups.

### 3.4. Stage-Specific RDA Identifying Physicochemical Factors Associated with Prokaryotic Succession and Butyric/Caproic Acid Accumulation

**Figure 8:**
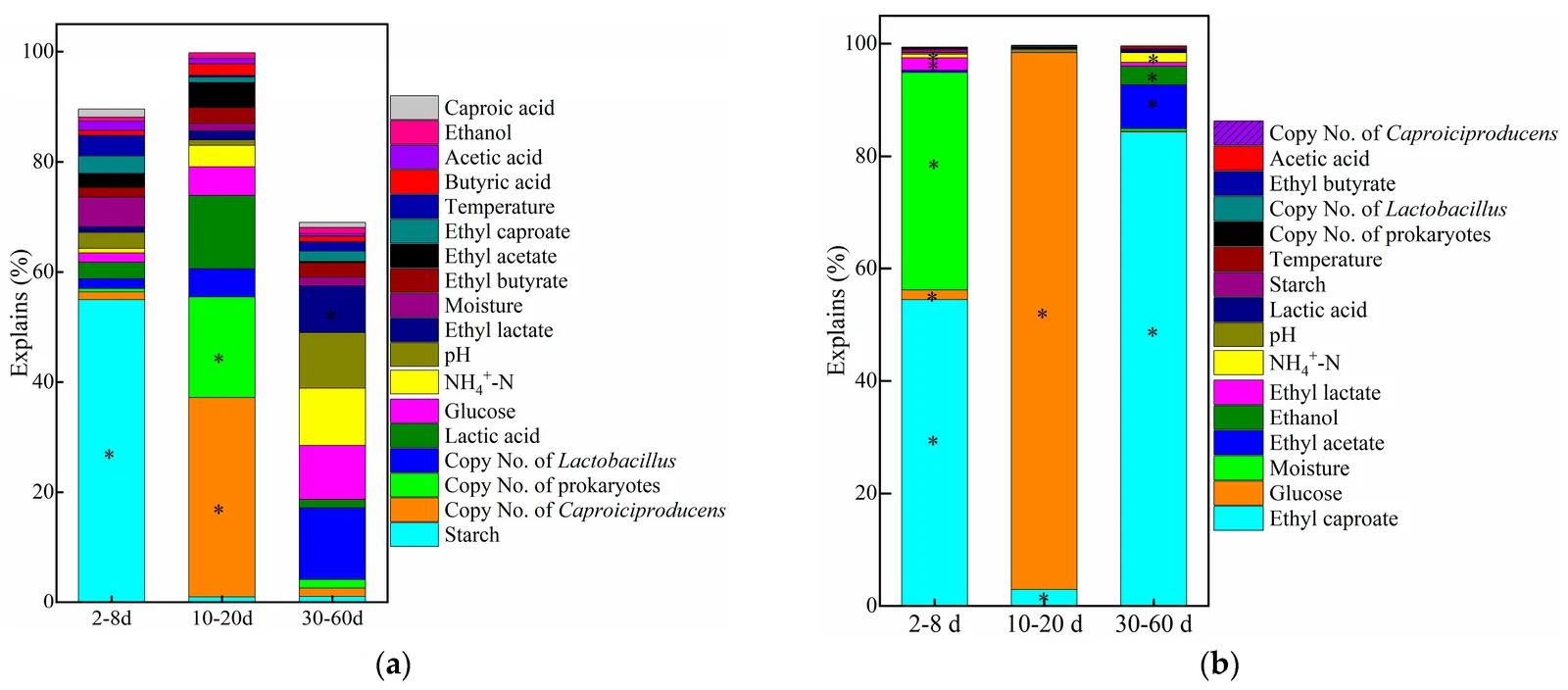
Stacked bar charts showing the proportion of explained variation (%) by individual physicochemical factors in redundancy analysis (RDA) for (**a**) prokaryotic community structure (with all physicochemical factors included as explanatory variables) and (**b**) the accumulation of butyric and caproic acids (with butyric and caproic acids excluded from the explanatory variables), across three fermentation stages (2–8 d, 10–20 d, and 30–60 d). Asterisks indicate statistically significant explanatory contributions of the corresponding factor (*p* < 0.05).

Based on the analysis of variations in microbial community structure and physicochemical parameters, stage-specific (2–8 d, 10–20 d, 30–60 d) RDA was performed to identify the key physicochemical factors associated with the succession of prokaryotic community structure (the primary contributors to butyric and caproic acids synthesis) and butyric and caproic acid accumulation (Figure 8, Figures S10 and S11; Files S3–S8).

Strong associations were observed between the two sets of explanatory variables and the response matrices. For prokaryotic community structure, the first two axes explained 85.66%, 97.99%, and 64.74% of variation during the early (2–8 d), middle (10–20 d), and late (30–60 d) stages, respectively, with strong species-environment correlations (r = 0.9569/0.9111, 1.0/1.0, and 0.8406/0.8446). For butyric and caproic acid accumulation (with the two acids excluded from explanatory variables), the first two axes explained 100% of variation—predominantly concentrated on Axis 1 (99.51%, 99.45%, and 99.79%, respectively)—with response-environment correlations remaining consistently strong (r = 0.9983/0.9813, 0.9997/0.9999, and 0.9995/0.9866). These results indicate that environmental conditions are significantly associated with two parallel dimensions of the UFG microbiota: community succession (the compositional dimension) and caproic acid metabolism (the functional dimension). During the early stage (2–8 d), starch (55%, *p* = 0.002) was identified as the dominant explanatory variables for prokaryotic community succession (Figure 8a and Figure S10a), whereas ethyl caproate (54.50%, *p* = 0.002) and moisture (38.70%, *p* = 0.002) were the dominant explanatory variables for butyric and caproic acid accumulation (Figure 8b and Figure S11a). These results indicate that substantial starch consumption and rapidly increasing moisture are key explanatory factors for prokaryotic community assembly and succession. Meanwhile, ethyl caproate—formed via caproic acid esterification—and moisture, which affects solute diffusion and microbial activity in the solid matrix, represent key explanatory factors associated with caproic acid synthesis, specifically the accumulation of butyric acid (an intermediate) and caproic acid. The large explanatory share of ethyl caproate should be interpreted cautiously: because esterification chemically links caproic acid and its ester, their statistical association in RDA may partly reflect this mathematical coupling rather than an ecological driver–response relationship (Figure 6f and Figure 7d). In both RDAs, the clear separation between aged- and new-pit UFG samples along Axis 1 reflected opposing gradients of starch/glucose/ethyl caproate (aged-pit UFG) versus moisture (new-pit UFG) (Figures S10a and S11a). Taken together, these findings suggest that butyric and caproic acid accumulation in aged-pit UFG appears to benefit from the sustained supply of carbon sources such as starch and glucose, which support microbial metabolism. By contrast, despite adequate moisture in new-pit UFG, the low levels of butyric and caproic acid accumulation likely stem from the microbial community’s inability to establish the complete metabolic chain from starch via glucose and lactic acid ultimately to butyric and caproic acids. High moisture may instead dilute local acid concentrations or promote competing metabolic pathways such as lactic acid fermentation [69].

In the middle stage (10–20 d), copy number of *Caproiciproducens* (36.20%, *p* = 0.008) and entire prokaryotic community (18.30%, *p* = 0.006) significantly explained the community variation (Figure 8a and Figure S10b); Whereas glucose (79.00%, *p* = 0.002) and ethyl acetate (17.30%, *p* = 0.002) were identified as the dominant explanatory variables for butyric and caproic acid accumulation (Figure 8b and Figure S11b). We speculate that the growth of *Caproiciproducens* (Figure 2) and the *Lactobacillus*-dominated prokaryotic community may have shaped the community structure [48,67,70,71]. Indeed, lactic acid alone explained 13.3% of the variation, albeit with marginal significance (*p* = 0.058). Meanwhile, the depletion of glucose as a carbon source, together with the growth stagnation of acetic acid-producing bacteria under low-pH conditions [63], and the reduced availability of acetic acid—serving as both a substrate and electron acceptor for chain elongation—due to its consumption for ethyl acetate synthesis [72], likely contributed to the stalled production of butyric and caproic acids during this stage. In both RDAs, clear separation between aged- and new-pit UFG samples along Axis 1 reflected opposing environmental gradients, with aged-pit UFG associated with higher pH, glucose, and starch, and new-pit UFG oriented toward higher moisture, ethanol, and lactic acid (Figures S10b and S11b). This configuration suggests that new-pit microbiota may exhibit relatively lower tolerance to elevated ethanol and lactic acid, whereas aged-pit microbiota, while capable of withstanding these stressors, appeared more strongly associated with carbon-source availability (glucose and starch) and pH. Indeed, glucose is recognized as a favorable carbon source for caproic acid synthesis [11], and pH constitutes a critical factor in the enrichment of caproic acid-producing bacteria [5,73].

Lastly, in the late stage (30–60 d), copy number of *Lactobacillus* (13.0%, *p* = 0.066), NH_4_^+^-N (10.4%, *p* = 0.074), pH (10.1%, *p* = 0.054), and glucose (10.4%, *p* = 0.052) mostly explained variation in community succession (Figure 8a and Figure S10c); whereas, ethyl caproate was the strongest explanatory variable for butyric and caproic acid accumulation (84.7%, *p* = 0.002), followed by additional significant explanatory variables including ethyl acetate (7.60%, *p* = 0.002), ethanol (3.20%, *p* = 0.002), NH_4_^+^-N (2.20%, *p* = 0.004), acetic acid (0.50%, *p* = 0.012), ethyl butyrate (0.40%, *p* = 0.016), temperature (0.30%, *p* = 0.022), and ethyl lactate (0.30%, *p* = 0.022) (Figure 8b and Figure S11c). This is presumably attributable to the distinctive metabolic transition toward the traditionally recognized stage of synthesis of various flavor compounds, including caproic acid [18,20,23,48,74]. Therefore, it is understandable that pH, which influences the growth of potential CPBs [5,73]; acetic acid as a substrate for caproic acid synthesis by CPBs; ethyl acetate as a factor affecting acetic acid availability; and *Caproiciproducens* and *Lactobacillus* as potential caproic acid-producing bacteria, collectively covaried with community succession [5,8,9,10,11,12,13,36,75]. Accordingly, variables associated with microbial proliferation (ethanol, NH_4_^+^-N), enzymatic activity (temperature), and caproic acid metabolism-associated substrates and products (ethyl acetate, ethyl butyrate, and ethyl caproate) served as key explanatory variables. Notably, the copy numbers of *Caproiciproducens* (14.6%) and *Lactobacillus* (8.3%) emerged as significant explanatory factors, suggesting that although *Caproiciproducens* remained at relatively low abundance in the UFG, it may play a structurally important role in the late-stage microbial community [29,33]. The RDA biplots revealed the most pronounced inter-pit divergence throughout fermentation (Figures S10c and S11c). New-pit UFG samples were associated with lactic acid, pH, residual starch, moisture, and temperature, indicating a persistent lactic acid-dominated state with limited progression toward chain elongation. In contrast, aged-pit UFG samples aligned with acetic acid, glucose, NH_4_^+^-N, and the accumulation of ethyl acetate, ethyl butyrate, and ethyl caproate, as well as the abundance of *Caproiciproducens*, indicating a mature metabolic network characterized by active acetic acid turnover and esterification.

In summary, the stage-resolved RDA identified key physicochemical parameters associated with both prokaryotic community succession and caproic acid metabolism in UFG. However, because RDA captures correlative rather than causal relationships, these associations require experimental validation to establish the underlying mechanisms.

### 3.5. Genus-Level Correlations with Caproic Acid Biosynthesis and Major Ethyl Esters: Aged vs. New Pits

#### 3.5.1. Genus-Level Correlations with Caproic Acid Biosynthesis: Aged vs. New Pits

**Figure 9:**
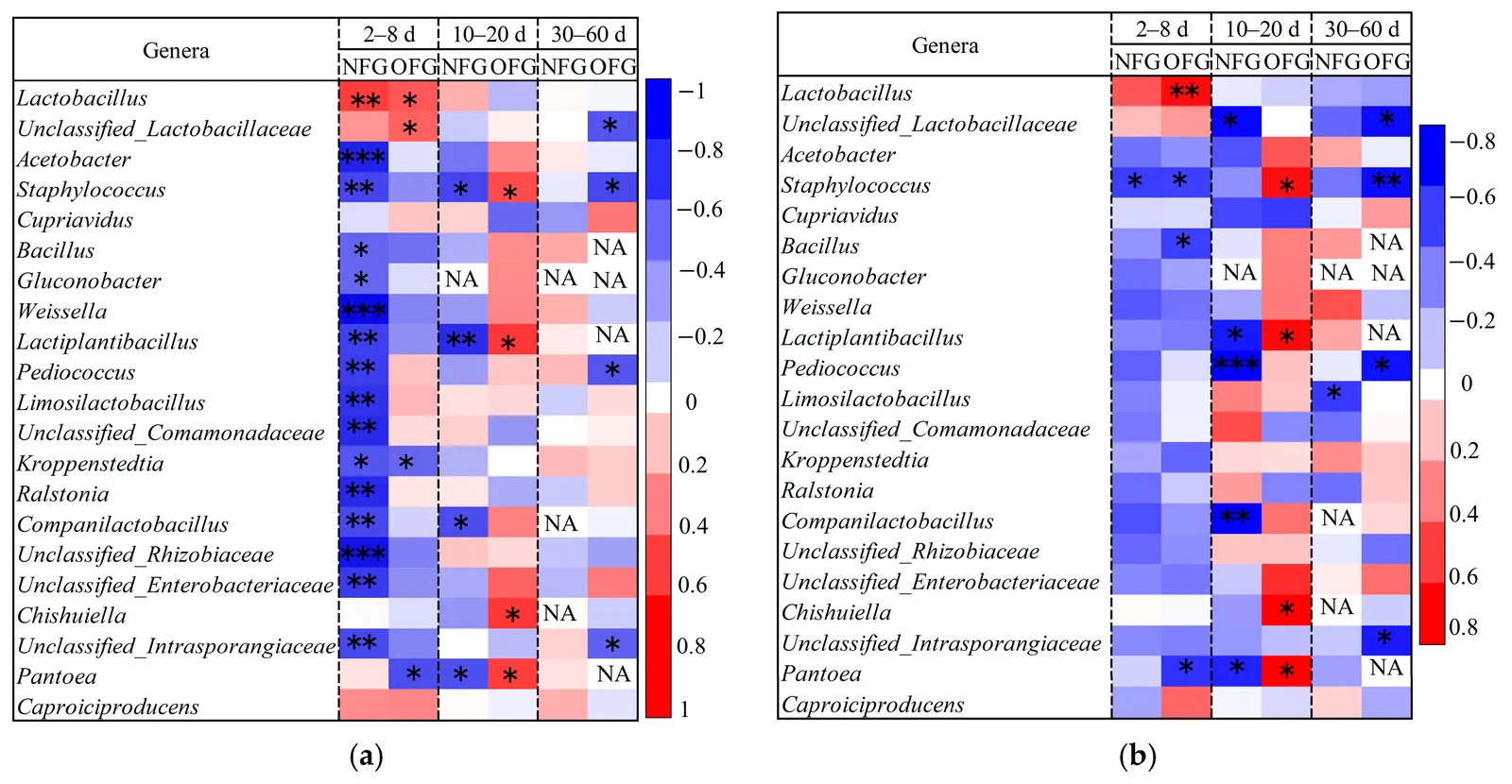
Pearson correlations between bacterial genera (dominant genera and the non-dominant traditional CPB genus *Caproiciproducens*) and (**a**) butyric acid (intermediate) and (**b**) caproic acid (product) of caproic acid synthesis in the UFG of new and aged pits across three fermentation stages: early (2–8 d), mid (10–20 d), and late (30–60 d). Asterisks denote significant differences between new and aged pits at corresponding time points (*, 0.01 < *p* < 0.05; **, 0.001 < *p* < 0.01; ***, *p* < 0.001). NA indicates missing correlation values due to zero values in the samples.

Given that all currently recognized CPBs are prokaryotes [3,5,8,9,10,11,12,13,14,15,16,17], Pearson correlation analysis was performed to assess the relationships between copy abundances of prokaryotic genera and concentrations of butyric and caproic acids, thereby identifying genera potentially associated with these acids in the UFG of aged and new pits (Figure 9).

During the first stage (2–8 d), the initial phase of butyric and caproic acid synthesis (Figure 6e,f), *Lactobacillus*, *Unclassified Lactobacillaceae*, and/or traditional CPB genus *Caproiciproducens* showed positive with butyric and caproic acids. A greater number of genera in new-pit UFG exhibited negative correlations with butyric and caproic acids, whereas in aged-pit UFG these negative correlations were attenuated or shifted toward positive, as exemplified by *Acetobacter*, *Staphylococcus*, *Cupriavidus*, *Pediococcus*, *Limosilactobacillus*, *Ralstonia*, *Weissella*, *Lactiplantibacillus* etc. In addition to the traditional CPB genus *Caproiciproducens* [5,8], some of these genera have been implicated in organic acid metabolism in previous studies, such as *Pediococcus*, *Weissella*, *Lactiplantibacillus* [76,77,78]. The above difference between aged- and new-pit UFG suggests that the early microbial community in new-pit UFG may still be undergoing environmental adaptation or interspecific competition, whereas the relatively mature aged-pit community may have established a more synergistic metabolic network for caproic acid synthesis—an inference that is further examined by the genus-level co-occurrence network analysis below (Section 3.6). This pattern is consistent with the higher synthesis levels of butyric and caproic acids in aged-pit UFG (Figure 6e,f). In addition, certain genera exhibited consistent positive correlations with both butyric and caproic acids across both aged- and new-pit UFG, including *Lactobacillus*, unclassified *Lactobacillaceae*, and *Cupriavidus*, suggesting that some may represent hitherto unidentified potential CPBs, or may engage in synergistic metabolic interactions with butyric acid-producing bacteria (BPBs) or CPBs. It is currently known that LAB and BPBs or CPBs exhibit synergistic metabolic interactions [56,79]. Furthermore, *Ligilactobacillus acidipiscis* YHS1A (16S rRNA gene accession No. PQ626723.1), isolated in our laboratory, was found to produce butyric acid and trace caproic acid [36]. In addition, whether the numerous potential non-traditional CPBs contribute to caproic acid synthesis directly, indirectly through precursor supply, or via other synergistic mechanisms remains to be confirmed.

During the second stage (10–20 d), as butyric and caproic acid concentrations declined and subsequently stabilized (Figure 6e,f), the majority of genera that had been positively correlated with these acids in aged-pit UFG during 2–8 d continued to exhibit positive correlations. In contrast, these correlations shifted to negative in new-pit UFG, as exemplified by *Acetobacter*, *Staphylococcus*, *Bacillus*, *Gluconobacter*, *Weissella*, *Lactiplantibacillus*, *Pediococcus*, *Companilactobacillus*, *uncultured Enterobacteriaceae*, *Chishuiella*, and *Pantoea*. We speculate that during this stage, potential CPBs and their synergistic partners in aged-pit UFG decreased in parallel, preserving stable positive correlations. This synchronized response is consistent with the putative acid-tolerance and metabolic interdependence of the aged-pit community. In contrast, the aforementioned genera in new-pit UFG may lack adaptive mechanisms to high lactic acid and ethanol stress, and have not yet established synergistic metabolic relationships with potential CPBs, which may result in decoupled dynamics and thus weaker correlations.

During the third stage (30–60 d), correlation patterns exhibited weaker continuity with those observed in the preceding two stages, presumably attributable to shifts in substrate structure and metabolic modes. Furthermore, the inter-pit divergence was more pronounced, likely reflecting the greater disparity in microbial community structure between aged- and new-pit UFG during this phase (Figure 1a). Surprisingly, the known CPB genus *Caproiciproducens* in aged-pit UFG did not exhibit a significant positive correlation with either butyric or caproic acids, possibly because this genus grows poorly in UFG, particularly under its relatively low moisture conditions, and is actually better adapted to the surface pit mud or lower-layer fermented grains [29].

#### 3.5.2. Genus-Level Correlations with Major Ethyl Esters: Aged vs. New Pits

**Figure 10:**
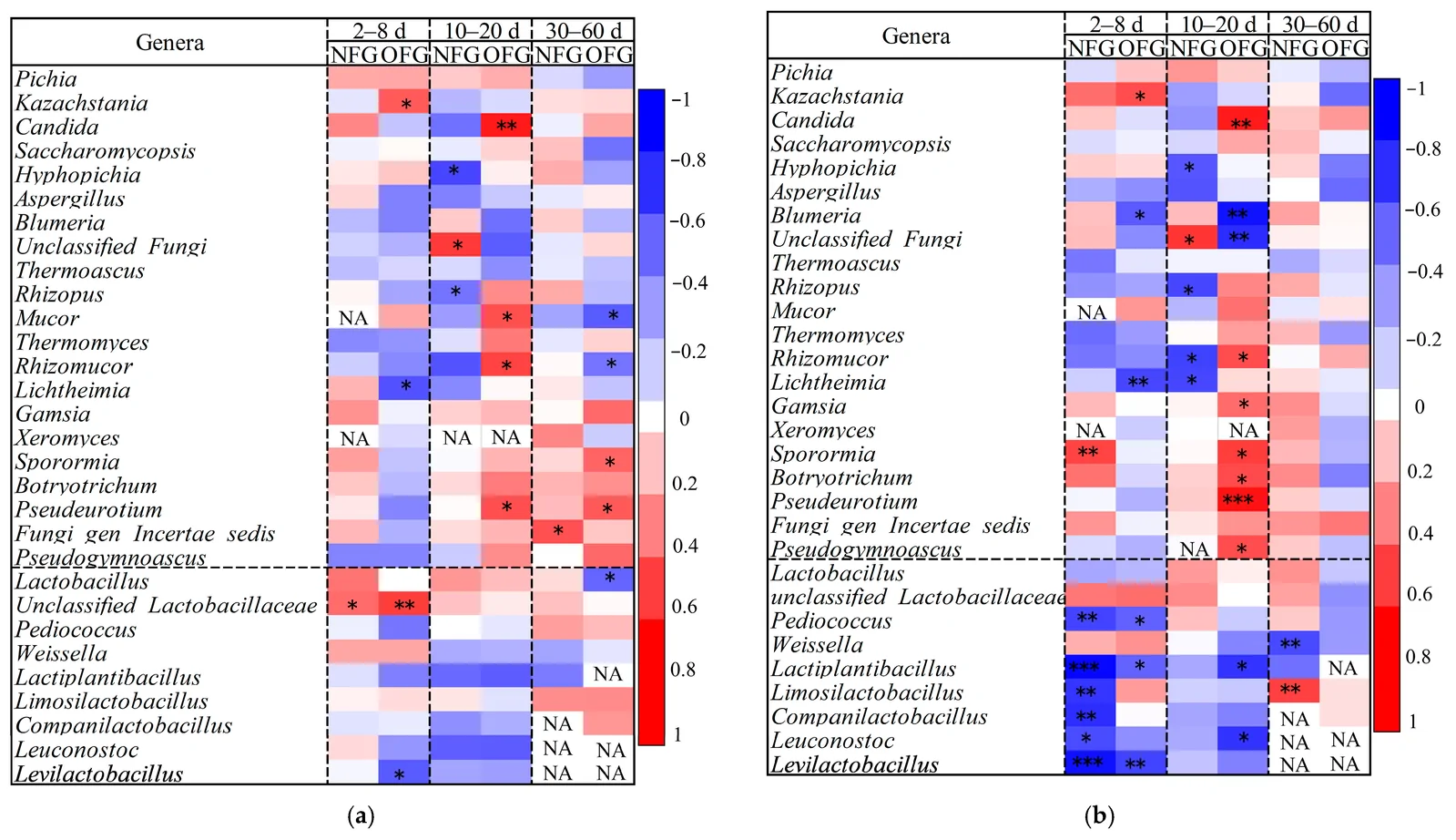

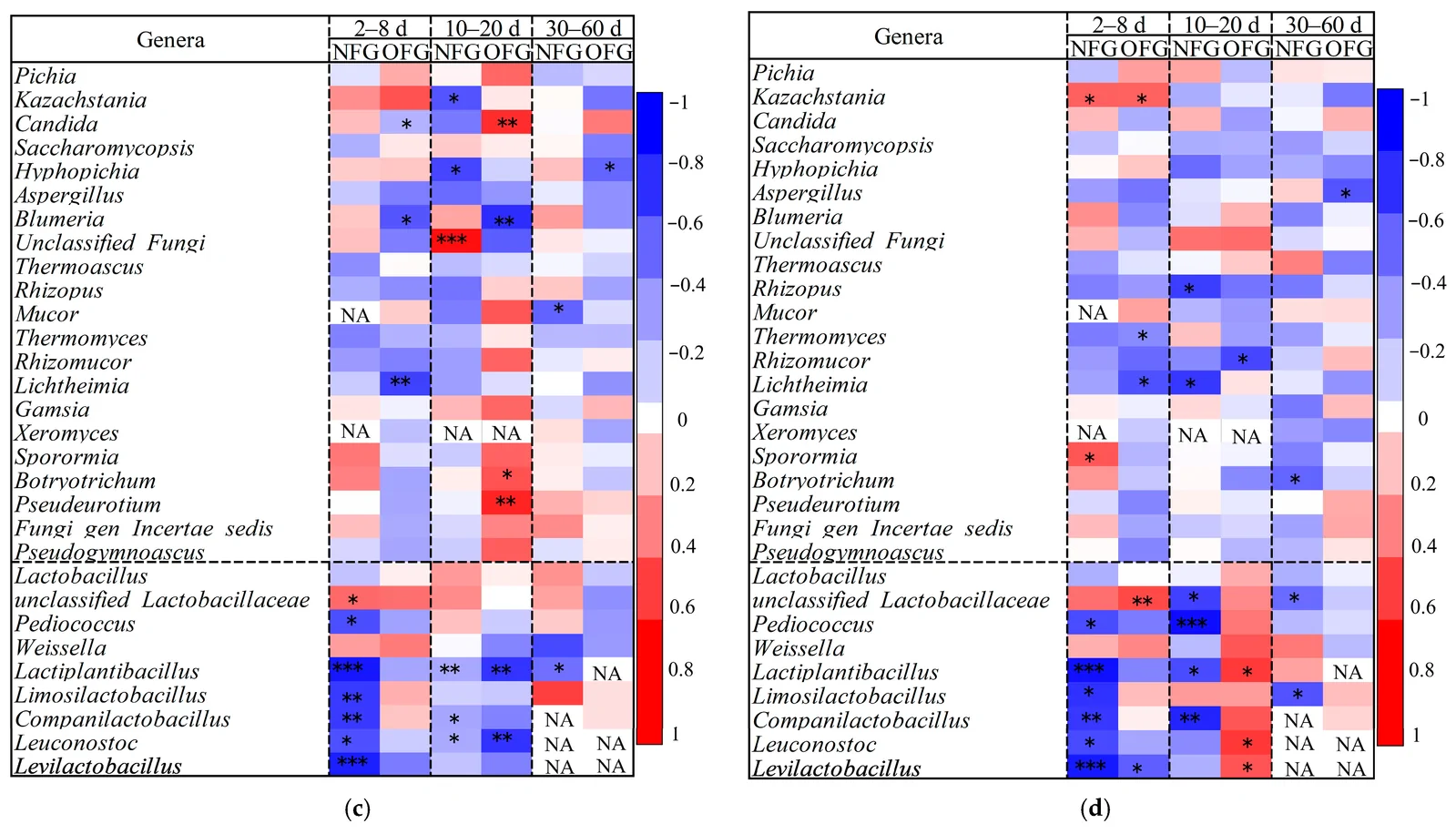
Correlations between potential esterification genera and (**a**) ethyl lactate, (**b**) ethyl acetate, (**c**) ethyl butyrate, and (**d**) ethyl caproate in the UFG of new and aged pits across three fermentation stages: early (2–8 d), mid (10–20 d), and late (30–60 d). Spearman correlations were calculated for fungal genera, and Pearson correlations for LAB genera. Asterisks denote significant differences between new and aged pits at corresponding time points (*, 0.01 < *p* < 0.05; **, 0.001 < *p* < 0.01; ***, *p* < 0.001). NA indicates missing correlation values due to zero values in the samples.

Given that fungi and LAB are recognized as the principal contributors of ester synthesis across fermented food systems [80] and in CSFB in particular [23,24], and that esterification levels vary among different-age pits [25], this section co-examines the correlations of fungal genera and LAB genera with the four esters, aiming to identify genera with potential esterification functions. Generally, the correlations exhibited significant stage-specific patterns. Notably, aged-pit UFG harbored a greater number of genera significantly positively correlated with the four esters than did new-pit UFG (Figure 10), a trend generally consistent with previous findings [23,25], although the specific genera involved differed. Detailed analysis of these genera is provided in subsequent sections.

Genera positively correlated with ethyl lactate, ethyl acetate, ethyl butyrate and ethyl caproate exhibited distinct stage-specific patterns and differential bacterial/fungal characteristics (Figure 7 and Figure 10). Specifically, ethyl lactate correlations were relatively limited during the early stage (2–8 d), represented by *Unclassified Lactobacillaceae* and *Kazachstania*, but intensified during the mid (10–20 d) and late (30–60 d) stages, with *Candida*, *Mucor*, *Rhizomucor*, and *Pseudeurotium* dominating the middle stage and *Sporormia*, *Pseudeurotium*, and *Fungi_gen_Incertae_sedis* prevailing in the late stage. Ethyl acetate correlations were primarily bacterial during the early stage (*Weissella* and *unclassified Lactobacillaceae*) and predominantly fungal during the middle stage (*Candida*, *Rhizomucor*, *Sporormia*, *Botryotrichum*, *Pseudeurotium*, and *Pseudogymnoascus*). Notably, aged-pit UFG harbored significantly more correlated genera than new-pit UFG for both esters. Genera positively correlated with ethyl butyrate and ethyl caproate were primarily found during the early (2–8 d) and mid (10–20 d) stages of rapid ester accumulation. During the early stage, ethyl butyrate correlations were primarily observed among bacterial genera, including *Weissella* and *unclassified Lactobacillaceae*, whereas ethyl caproate correlations were observed among fungal genera (*Kazachstania* and *Sporormia*) as well as *Lactobacillus*. During the mid stage, aged-pit UFG harbored significantly more correlated genera than new-pit UFG for both esters: ethyl butyrate was dominated by *Candida*, *Botryotrichum*, and *Pseudeurotium* in aged-pit UFG but by *unclassified Fungi* in new-pit UFG; ethyl caproate was predominantly associated with LAB genera, including *Lactiplantibacillus*, *Leuconostoc*, and *Levilactobacillus*. Notably, aged-pit UFG harbored significantly more genera positively correlated with the four esters than did new-pit UFG, suggesting that the greater metabolic network integrity associated with ecosystem maturity may contribute to the observed differences in fermentation outcomes between pit types, rather than the mere presence or absence of individual species or substrates. These findings partially align with previous studies [24,25,31,81], although some earlier reports identified *Pichia*—rather than *Candida* as found in this study—as the yeast genus most strongly associated with esters. It should be noted that the correlations reported in this section represent statistical associations; the identified genera are candidate esterification-associated taxa whose functional roles require strain-level validation.

### 3.6. Genus-Level Co-Occurrence Networks: Aged vs. New Pits

#### 3.6.1. Prokaryotic Co-Occurrence Networks: Aged vs. New Pits

**Figure 11:**
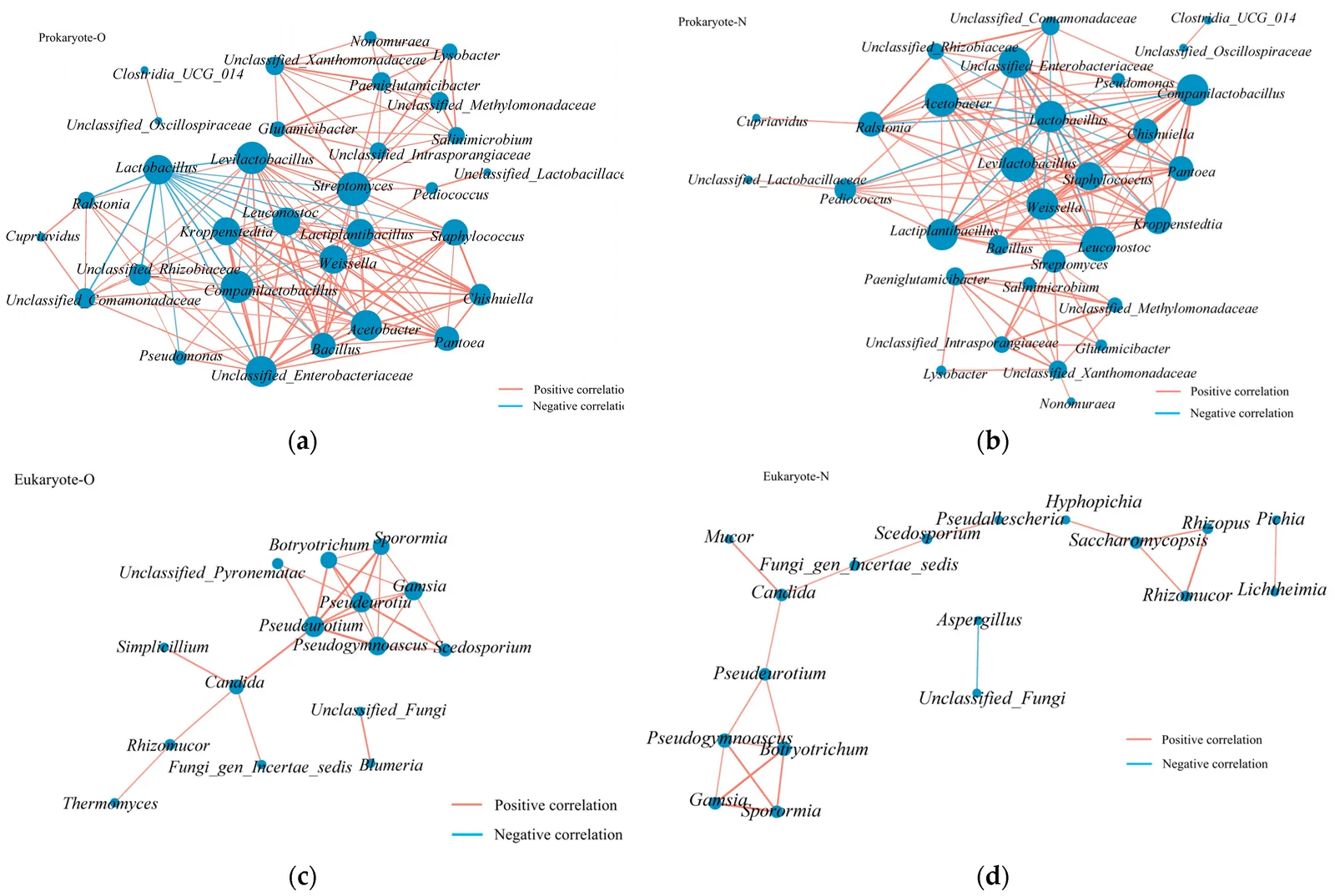
Co-occurrence networks among representative genera of the UFG microbiota based on Spearman’s rank correlation analysis. (**a**,**b**) Prokaryotic networks of aged-pit (**a**) and new-pit (**b**) UFG; (**c**,**d**) eukaryotic networks of aged-pit (**c**) and new-pit (**d**) UFG. Networks were constructed separately for prokaryotic and eukaryotic communities from genus-level abundance data, and only strong and significant correlations (Spearman’s r > 0.6 or r < −0.6, *p* < 0.05) were retained as edges. Nodes represent genera (sized by mean relative abundance), and edges represent significant correlations between genera (red, positive; blue, negative), with edge thickness proportional to the absolute correlation coefficient. Networks were visualized using Cytoscape (v3.10.2).

Co-occurrence networks of aged-pit and new-pit UFG prokaryotic communities shared 31 nodes but differed in connectivity (Figure 11a,b; File S1). Both networks were dominated by positive edges (89.9% and 88.7%, respectively; all edges FDR < 0.05), consistent with the prevalence of cooperative interactions within baijiu fermentation consortia [19], and in both, all 15 negative edges were exclusively associated with *Lactobacillus*, identifying the dominant acid producer as a universal competitive hub—in line with the well-documented dominance and acid resistance of lactobacilli in CSFB [36,70]. Relative to the new-pit network, the aged-pit network was more structured, with more edges (148 vs. 133), higher density (0.318 vs. 0.286), clustering coefficient (0.744 vs. 0.665) and modularity (0.351 vs. 0.297; five vs. four modules), and shorter average path length (1.985 vs. 2.209). Of 161 unique edges, 120 were shared (Jaccard similarity = 0.745), indicating a conserved co-occurrence backbone, whereas 28 aged-pit-specific and 13 new-pit-specific edges reflected pit-dependent reorganization, echoing previous findings that prokaryotic communities differ markedly between old and young pits [2,42] and undergo age-dependent succession in both pit mud and FG [3]. *Streptomyces* showed the highest betweenness centrality in both networks (0.383 and 0.370), bridging the actinomycete and core modules despite being negatively correlated with *Lactobacillus*. Thus, competitive exclusion by *Lactobacillus* appears to be a pit-age-independent organizing principle, whereas pit aging fine-tunes the prokaryotic network toward greater connectivity and compartmentalization, consistent with the intensified environmental filtering (e.g., pH-driven selection) reported to shape pit-microbial communities [72].

#### 3.6.2. Eukaryotic Co-Occurrence Networks: Aged vs. New Pits

Eukaryotic co-occurrence networks were much smaller but exhibited a stronger pit-age effect (Figure 11c,d; File S2). The aged-pit network (15 nodes, 26 edges) consisted of a single large component (13 nodes) organized around a densely interconnected ascomycete core module (*Botryotrichum*, *Gamsia*, *Pseudallescheria*, *Pseudeurotium*, *Pseudogymnoascus*, *Scedosporium*, *Sporormia*, and *unclassified Pyronemataceae*; 20 internal edges) and contained no negative edges, with *Candida* and *Pseudeurotium* serving as the principal bridges (betweenness = 0.407 each)—the prominence of *Candida* being consistent with its recognized role as an aroma-producing yeast in baijiu [49,50], and the dominance of filamentous ascomycetes agreeing with reports that fungi govern stage-specific metabolism in the pit microecology [24] and that fungal community composition in pit mud varies with pit age [53,54]. In contrast, the new-pit network (18 nodes, 19 edges) was fragmented into four disconnected subnetworks (10, 4, 2, and 2 nodes) with only half the network density (0.124 vs. 0.248); mucoralean genera and yeasts (*Rhizopus*–*Saccharomycopsis*–*Rhizomucor*–*Hyphopichia*; *Pichia*–*Lichtheimia*) formed loose peripheral associations—Rhizopus and Mucor being typical pioneer saccharifying fungi in cereal fermentations [51]—and the single negative edge linked *Aspergillus* with unclassified Fungi. Only 11 edges were shared between the two networks (Jaccard similarity = 0.324), concentrated in the *Gamsia–Sporormia*–*Botryotrichum*–*Pseudogymnoascus* association and the *Candida*–*Pseudeurotium* bridge, suggesting that pit aging consolidates this conserved ascomycete backbone into an integrated, cooperative network.

#### 3.6.3. Pit-Age Effects Are Stronger on Eukaryotic than Prokaryotic Interaction Networks

Comparing the magnitude of pit-age effects between the two kingdoms indicates that eukaryotic co-occurrence networks differentiate more strongly between aged and new pits than prokaryotic networks. Differential responses of prokaryotic and eukaryotic microbiomes to the same environmental gradient have also been documented in other ecosystems [39]. Although both kingdoms changed in the same direction (aged-pit networks being more connected and organized), the relative differences were consistently larger in eukaryotes: network density doubled (0.248 vs. 0.124, +100%), edges increased by 37%, clustering coefficient by 35%, and average path length shortened by 18%, whereas the corresponding prokaryotic changes were modest (density +11%, edges +11%, clustering +12%, path length −10%). More importantly, the eukaryotic topology shifted qualitatively—from a single integrated component in aged pits to four disconnected subnetworks in new pits—while the prokaryotic network retained the same overall architecture, with 74.5% of edges shared between pits (Jaccard similarity = 0.745) compared with only 32.4% in eukaryotes (Jaccard similarity = 0.324). Notably, eukaryotic co-occurrence networks exhibited marked fragmentation in the new pits, where four distinct sub-networks formed, in contrast to the single, integrated unit observed in the aged pits. These results suggest that pit age exerts a quantitative, fine-tuning effect on the prokaryotic community, whose interaction backbone (including the *Lactobacillus*-centered competitive structure) is already established in young pits, but a qualitative, restructuring effect on the eukaryotic community, whose interaction network is consolidated only in mature pits—in agreement with the view that long-term pit usage drives progressive specialization of the pit microbiota [28] and that older fermentation systems harbor more mature, flavor-relevant consortia [4]. Several non-exclusive mechanisms may underlie this differential response of the two communities. First, organic acids (butyric, caproic, and lactic acids) accumulate to characteristic levels in aged pits, which may both select for acid-tolerant fungal taxa and act as substrates or signaling molecules that foster cross-species metabolic coupling. Second, the longer colonization history and higher diversity of aged pits (Figure 3b) may allow rare fungal taxa to establish persistent populations, thereby increasing opportunities for niche complementarity and mutualistic associations. Third, the matured pit microenvironment—characterized by stable temperature, moisture, and anaerobic gradients, together with the established pit-mud microbial reservoir—may provide a more homogeneous and continuous habitat that favors network integration. We emphasize that these mechanisms remain testable hypotheses generated from observational data: because correlation-based co-occurrence reflects statistical association rather than direct ecological interaction [57], and given the small size of the eukaryotic networks (15–18 nodes) and the known limitations of ITS-based fungal identification [45,82], the observed differences should be interpreted as directional trends rather than precise effect sizes; distinguishing among these mechanisms will therefore require manipulative experiments.

Several limitations should be noted. First, although liquid-system spike-recoveries confirmed quantitative ester recovery, matrix-matched recovery in the solid UFG could not be assessed; the reported concentrations are therefore best regarded as semi-quantitative estimates, with emphasis on patterns and relative differences between pit groups. Second, no enzyme activity assays, strain-level functional validation, or metabolic flux measurements were performed; functional interpretations derived from PICRUSt2 predictions and correlations therefore represent hypotheses rather than demonstrated activities, and the pooled-time-point correlations may partly reflect shared temporal trends. Finally, the study was conducted at a single production facility during one 60-day cycle with a fixed grain formulation, without sensory or distilled-product analysis; the conclusions thus represent associations observed in the investigated pits, and their generalizability to other production systems remains to be verified.

## 4. Conclusions

This study systematically compared the temporal dynamics of microbial communities, physicochemical factors, and key metabolites associated with caproic acid synthesis and esterification in aged- and new-pit UFG during CSFB fermentation. The main conclusions are as follows:

(1) Pit age exerted contrasting effects on the two microbial domains: prokaryotic succession was largely conserved between aged and new pits, whereas the eukaryotic community was qualitatively restructured—aged-pit UFG being enriched in yeasts associated with butyric and caproic acid ester formation, and new-pit UFG harboring more taxa of as-yet-unclear fermentative relevance. UFG fermentation resolved into three ecological stages—early community reconstruction with initial butyric/caproic acid synthesis; middle stabilization with stalled chain elongation and partial acid degradation; and late secondary butyric/caproic acid synthesis—with the pit-age disparity becoming most pronounced in the late stage.

(2) New-pit UFG exhibited weaker acid production, consistent with a putatively incomplete and inefficient chain-elongation pathway, lacking the full three-phase dynamics and yielding lower final levels of most of the major ethyl esters. The accumulation of ethyl butyrate despite negligible free butyric acid suggests that the limited butyrate pool may be preferentially channeled toward esterification rather than chain elongation.

(3) Stage-resolved RDA models revealed shifting, stage-specific associations: prokaryotic community assembly was explained by starch and moisture at early stage, pH alone at mid stage, and a multi-factor network (pH, acetic acid, ethyl acetate, and *Caproiciproducens* and *Lactobacillus* abundance) in late stage fermentation; meanwhile, butyric/caproic acid accumulation was associated with ethyl caproate and moisture at early stage, with glucose and ethyl acetate at mid stage—coinciding with the stagnation of chain elongation—and predominantly by ethyl caproate at late stage.

(4) Correlation analyses identified candidate functional genera beyond traditional chain-elongating bacteria: several LAB genera (including *Lactobacillus*, *Pediococcus*, and *Weissella*) consistently correlated positively with butyric and/or caproic acids, suggesting previously unrecognized potential caproic acid producers or their synergistic partners, whereas the known CPB genus *Caproiciproducens* showed no significant correlation—likely reflecting its poor adaptation to the low-moisture UFG environment. New-pit UFG harbored markedly fewer genera correlated with ethyl butyrate and ethyl caproate than aged-pit UFG, with *Candida* and *Lactobacillus* identified as potential esterification contributors. Collectively, these candidate genera provide potential inoculation targets for new-pit improvement.

(5) Genus-level co-occurrence networks corroborated the maturity-dependent organization of the UFG microbiota. The aged-pit networks were more densely interconnected and modular than their new-pit counterparts: the aged-pit eukaryotic network formed an integrated, cooperative unit, whereas its new-pit counterpart was fragmented into disconnected subnetworks, suggesting that cooperative interactions among microbial taxa have yet to be established in this immature ecosystem. Moreover, pit-age effects were markedly stronger on eukaryotic than on prokaryotic networks, indicating that prokaryotic interactions are quantitatively fine-tuned whereas eukaryotic interactions are qualitatively restructured during pit maturation.

Collectively, these findings indicate that the disparity between aged and new pits is associated with the integrity of the maturity-driven metabolic and ecological networks—encompassing both substrate–product–ester coupling and cooperative connectivity among genera—rather than with the mere presence or absence of specific species or substrates. Accordingly, improving new-pit fermentation outcomes may benefit more from community-level metabolic synergy and synthetic-microbiome engineering than from single-strain supplementation. It should be emphasized that no artificially constructed microbial consortia were assembled or tested in this study; the performance and ecological suitability of such consortia therefore remain to be evaluated experimentally. As an observational, single-cycle study, these conclusions should be regarded as hypotheses guiding future manipulative experiments.

## Data Availability

The original Data presented in this study are included in Section 2.6 and “Supplementary Materials”. Further inquiries can be directed to the corresponding authors.

## Supplementary Materials

The following supporting information can be downloaded at https://www.mdpi.com/article/10.3390/foods15213975/s1. Figure S1: Schematic illustration of the sampling method for upper fermenting grains (UFG), samples were collected from the interval between the Huangshui line and the covering FG layer.; Figure S2: Temporal succession of prokaryotic communities at the phylum level in upper fermented grains (UFG) from new and aged pits during the 60-day fermentation; Figure S3: Temporal succession of prokaryotic communities at the family level in upper fermented grains (UFG) from new and aged pits during the 60-day fermentation; Figure S4: Temporal succession of eukaryotic communities at the phylum level in upper fermented grains (UFG) from new and aged pits during the 60-day fermentation; Figure S5: Temporal succession of eukaryotic communities at the family level in upper fermented grains (UFG) from new and aged pits during the 60-day fermentation; Figure S6: Temporal dynamics of Pearson correlations between PICRUSt2-predicted fatty acid biosynthesis gene abundance and the absolute abundance of dominant prokaryotic genera in new (NFG) and aged-pit UFG (OFG), NA indicates missing correlation coefficients due to zero values in samples; Figure S7: Dynamics of temperature (a), starch (b), NH_4_^+^-N(c), moisture (d), and pH (e) of UFG during fermentation from new and aged pits. Data are presented as means ± SD (*n* = 3 independent pits per group; error bars represent the standard deviation calculated across the three pits). Asterisks indicate significant differences between new and aged pit samples at the corresponding time points (*p* < 0.05, ANOVA); Figure S8: Absolute abundances of the total prokaryotic community and the dominant *Lactobacillus* in UFG from new and aged pits during fermentation. Data are presented as means ± SD (*n* = 3 independent pits per group). Asterisks above the data points indicate significant differences (*p* < 0.05, ANOVA) between the total 16S rRNA gene copy numbers of the prokaryotic community in new-pit UFG (“Prokaryotic community NFG”) and those of the dominant genus *Lactobacillus* in new-pit UFG (“*Lactobacillus* NFG”); asterisks below the data points indicate significant differences (*p* < 0.05, ANOVA) between the total 16S rRNA gene copy numbers of the prokaryotic community in aged-pit UFG (“Prokaryotic community OFG”) and those of the dominant genus *Lactobacillus* in aged-pit UFG (“*Lactobacillus* OFG”); Figure S9: Dynamics of the total molar amounts of the four major acids and their corresponding ethyl esters in UFG from new and aged pits during fermentation. The molar sum of each acid–ester pair was calculated to reflect the total molar quantity of the acid synthesized during fermentation. (a) Lactic acid + ethyl lactate; (b) Acetic acid + ethyl acetate; (c) Butyric acid + ethyl butyrate; (d) Caproic acid + ethyl caproate. Data are presented as means ± SD (*n* = 3 independent pits per group); Figure S10: RDA ordination biplots showing the relationships between prokaryotic community structure and physicochemical attributes in new and aged pit UFG across three fermentation stages: 2–8 d (a), 10–20 d (b), and 30–60 d (c). The arrows indicate the direction and magnitude of physicochemical attributes associated with prokaryotic community structures; Figure S11. RDA ordination biplots showing the relationships between physicochemical factors excluding butyric and caproic acids and the accumulation of butyric and caproic acids in new and aged pit UFG across three fermentation stages: 2–8 d (a), 10–20 d (b), and 30–60 d (c). The arrows indicate the direction and magnitude of physicochemical attributes associated with prokaryotic community structures; Table S1: Raw physicochemical data of UFG samples from aged and new pits during fermentation; Table S2: Sample metadata (sample ID, pit age, sampling date) and fermentation process parameters (grain formulation, loading, Daqu dose, and recycled-grain information) for aged– and new–pit UFG samples.; Table S3: ASV table for 16S rRNA amplicon sequencing of UFG samples, with the corresponding NCBI accession numbers for the raw sequencing data of each sequencing subsample; Table S4: ASV table for ITS amplicon sequencing of UFG samples, with the corresponding NCBI accession numbers for the raw sequencing data of each sequencing subsample; Table S5: Original PICRUSt2 functional prediction output for 16S rRNA amplicon sequencing data, based on the KEGG (Kyoto Encyclopedia of Genes and Genomes) database; Table S6: Original PICRUSt2 functional prediction output for ITS amplicon sequencing data, based on the MetaCyc database; Table S7: qPCR-based total 16S rRNA gene copy numbers; File S1: Original R script for the Pearson correlation analysis between prokaryotic genera (qPCR-based absolute abundances) and metabolite concentrations; File S2: Original R script for the Spearman correlation analysis between eukaryotic genera (ITS-based relative abundances) and the metabolite concentrations; File S3: RDA analysis data for the prokaryotic community structure and physicochemical attributes of aged- and new-pit UFG samples during the early stage (2–8 d); File S4: RDA analysis data for the prokaryotic community structure and physicochemical attributes of aged- and new-pit UFG samples during the middle stage (10–20 d); File S5: RDA analysis data for the prokaryotic community structure and physicochemical attributes of aged- and new-pit UFG samples during the late stage (30–60 d); File S6: RDA analysis data for butyric/caproic acid accumulation and physicochemical factors (acids excluded) during early (2–8 d) stage; File S7: RDA analysis data for butyric/caproic acid accumulation and physicochemical factors (acids excluded) during middle (10–20 d) stage; File S8: RDA analysis data for butyric/caproic acid accumulation and physicochemical factors (acids excluded) during late (30–60 d) stage; File S9: Topological properties of the prokaryotic co-occurrence networks in aged-pit and new-pit UFG samples; File S10: Topological properties of the eukaryotic co-occurrence networks in aged-pit and new-pit UFG samples.

## References

[1] (2021). Baijiu Quality Requirements—Part 1: Nongxiangxing Baijiu.

[2] Zhang H., Meng Y., Wang Y., Zhou Q., Li A., Liu G., Li J., Xing X. (2020). Prokaryotic communities in multidimensional bottom-pit-mud from old and young pits used for the production of Chinese Strong-Flavor Baijiu. Food Chem..

[3] Tong Y., Jiang M., Li X., Deng B., Zhang S., Wang S., Liu M., Yu J., Song P. (2025). Age-dependent microbial succession and functional differentiation in pit mud and Zaopei of strong-flavor Baijiu. LWT-Food Sci. Technol..

[4] Xu S., Zhang M., Xu B., Liu L., Sun W., Mu D., Wu X., Li X. (2022). Microbial communities and flavor formation in the fermentation of Chinese strong-flavor Baijiu produced from old and new Zaopei. Food Res. Int..

[5] Zhang H., Wang X., Zhen L., Chang Q., Cui L., Xue Z.L. (2025). Composition and metabolite patterns of caproic acid-producing bacteria during pH-mediated pitmud-Huangshui co-fermentation based on multi-database annotation. Food Chem..

[6] Zhang H., Zhen L., Chang Q., Liu Y., Xu K., Wang X., Cui L., Wu Z., Xue Z. (2025). The effect of autochthonous caproic acid-producing consortia inoculation on the multi-dimensional prokaryotic succession of bottom pitmud used for the Chinese strong-flavor Baijiu fermentation. Curr. Res. Microbiol. Sci..

[7] Zhou H., Xu S., Xu B., Jiang C., Zhao E., Xu Q., Hong J., Li X. (2024). Effect of *Caproicibacterium lactatifermentans* inoculation on the microbial succession and flavor formation of pit mud used in Chinese Baijiu fermentation. Food Res. Int..

[8] Jin X., Wang H., Tian H., Hu Y., Peng N., Zhao S. (2025). *Caproiciproducens* converts lactic acid into caproic acid during Chinese strong-flavor Baijiu brewing. Int. J. Food Microbiol..

[9] Zhu X.Y., Tao Y., Liang C., Li X.Z., Wei N., Zhang W.J., Zhou Y., Yang Y.F., Bo T. (2015). The synthesis of n-caproate from lactate: A new efficient process for medium-chain carboxylates production. Sci. Rep..

[10] Zeng C.Y., Zeng X., Xia S., Ye G. (2024). *Caproicibacterium argilliputei* sp. nov., a novel caproic acid producing anaerobic bacterium isolated from pit clay. Int. J. Syst. Evol. Microbiol..

[11] Wang H., Gu Y., Zhou W., Zhao D., Qiao Z., Zheng J., Gao J., Chen X., Ren C., Xu Y. (2021). Adaptability of a caproate-producing bacterium contributes to its dominance in an anaerobic fermentation system. Appl. Environ. Microbiol..

[12] Wang H., Gu Y., Zhao D., Qiao Z., Zheng J., Gao J., Ren C., Xu Y. (2022). *Caproicibacterium lactatifermentans* sp. nov., isolated from pit clay used for the production of Chinese strong aroma-type liquor. Int. J. Syst. Evol. Microbiol..

[13] Dai M., Xu Y., Zhao L., Wu M., Ma H., Zhu L., Li W., Li X., Sun B. (2024). *Caproicibacter* sp. *BJN*0012, a potential new species isolated from cellar mud for caproic acid production from glucose. J. Biotechnol..

[14] Zhen L., Xu K.J., Cui L., Cui Z.Y., Liu Y., Wang X., Chang Q., Li J., Ren Y., Wang Y. (2026). Isolation, Metabolic Characteristics and Comparative Genomic Analysis of the Dominant Caproic Acid-Producing Bacterium *Caproicibacterium lactatifermentans* XB2 from Pit Mud of Baijiu. Food Sci..

[15] Liu C., Du Y., Zheng J., Qiao Z.W., Luo H.B., Zou W. (2022). Production of caproic acid by *Rummeliibacillus suwonensis* 3B-1 isolated from the pit mud of strong-flavor baijiu. J. Biotechnol..

[16] Luo H., Li T., Zheng J., Zhang K., Qiao Z., Luo H., Zou W. (2022). Isolation, identification, and fermentation medium optimization of a caproic acid-producing *Enterococcus casseliflavus* strain from pit mud of Chinese strong flavor baijiu ecosystem. Pol. J. Microbiol..

[17] Wang Y., Li B., Dong H., Huang X., Chen R., Chen X., Yang L., Peng B., Xie G., Cheng W. (2018). Complete genome sequence of *Clostridium kluyveri* JZZ applied in Chinese strong-flavor liquor production. Curr. Microbiol..

[18] Gao J., Liu G., Li A., Liang C., Ren C., Xu Y. (2021). Domination of pit mud microbes in the formation of diverse flavour compounds during Chinese strong aroma-type Baijiu fermentation. LWT-Food Sci. Technol..

[19] Qian W., Lu Z., Chai L., Zhang X., Li Q., Wang S., Shen C., Shi J., Xu Z. (2021). Cooperation within the microbial consortia of fermented grains and pit mud drives organic acid synthesis in strong-flavor Baijiu production. Food Res. Int..

[20] Ren Z., Chen Q., Tang T., Huang Z. (2025). Unraveling the water source and formation process of Huangshui in solid-state fermentation. Food Sci. Biotechnol..

[21] Xu Y., Zhao J., Liu X., Zhang C., Zhao Z., Li X., Sun B.G. (2022). Flavor mystery of Chinese traditional fermented Baijiu: The great contribution of ester compounds. Food Chem..

[22] Li L.S., Fan M., Xu Y., Zhang L., Qian Y., Tang Y., Li J., Zhao J., Yuan S., Liu J. (2023). Comparative Analysis of Volatile Flavor Compounds in Strongly Flavored Baijiu under Two Different Pit Cap Sealing Processes. Foods.

[23] Li Z., Chen Y., Zheng J., Su J., Lu Y., Xia Y., Luo H. (2025). From fungi to lactic acid bacteria: Dominant microorganisms govern stage-specific esters biosynthesis in pit microecology of strong-flavor baijiu. Food Res. Int..

[24] Yi X., Xia H., Huang P., Ma S., Wu C. (2024). Exploring community succession, assembly patterns, and metabolic functions of ester-producing-related microbiota during the production of Nongxiangxing baijiu. Foods.

[25] Zhang J., Duan Y., Lin Y., Chen J., Cheng J., Song C., Zuo J., Zhang S., Zuo Y. (2025). Effects of pits of different ages on ethyl acetate and its metabolism-related microorganisms during strong-flavor Baijiu fermentation. Front. Microbiol..

[26] Li Z., Zhao C., Mao Z., Zhang F., Dong L., Song C., Chen Y., Fu X., Ao Z., Xiong Y. (2025). Structure and metabolic function of spatiotemporal pit mud microbiome. Environ. Microbiome.

[27] Zhu K., Xiao Q., Yuan S., Liu J., Shang H., Guo M., Zhao J. (2025). The relationship between microbial diversity and the physicochemical characteristics of pit mud of Strong-flavor Baijiu. Sci. Rep..

[28] Zeng Y., Zhong X., Chai L., Zhang X., Lu Z., Liu G., Tu T., Lu L., Zhang R., Yu H. (2025). Prokaryotic evolution shapes specialized communities in long term engineered pit mud ecosystem. npj Biofilms Microbiomes.

[29] Xu H., Kong W., Tang Q., Fan K., Liu M., Mo K., Xu Z., Zhang W. (2026). Analysis of microbiome succession and metabolome dynamics in Jiupei during Chinese strong-flavor Baijiu fermentation. Food Res. Int..

[30] Xu H., Tang Q., Fan K., Liu M., Wang L., Mo K., Xu Z., Zhang W. (2025). Microbiome-metabolome correlations across vertical stratification in Jiupei of strong-flavored baijiu fermentation. Food Res. Int..

[31] Huang Z., Zeng B., Deng J., Ren Z., Xie J., Wei C. (2024). Succession of microbial community structure in fermented grains during the fermentation of strong-flavor Baijiu and its impact on the metabolism of acids, alcohols, and esters. Food Sci. Biotechnol..

[32] Liu S., Ren D., Qin H., Yin Q., Yang Y., Liu T., Zhang S., Mao J. (2023). Exploring major variable factors influencing flavor and microbial characteristics of upper jiupei. Food Res. Int..

[33] Hu X., Tian R., Wang K., Cao Z., Yan P., Li F., Li X., Li S., He P. (2021). The prokaryotic community, physicochemical properties and flavors dynamics and their correlations in fermented grains for Chinese strong-flavor Baijiu production. Food Res. Int..

[34] Xu S., Zhou H., Xu B., Liu W., Hu W., Xu Q., Hong J., Liu Y., Li X. (2024). Environmental microbiota and its influence on microbial succession and metabolic profiles in baijiu fermentation across three distinct-age workshops. LWT-Food Sci. Technol..

[35] Liu X., Ma D., Yang C., Yin Q., Liu S., Shen C., Mao J. (2023). Microbial community succession patterns and drivers of Luxiang-flavor Jiupei during long fermentation. Front. Microbiol..

[36] Zhen L., Xu K., Xing X., Xue Z., Wang Y., Liu Y., Wang B., Wang T., Wang X., Zhang H. (2025). *Lactobacilli synthesize* butyric and caproic acids in strong-flavor Baijiu fermentation revealed by in situ, genomic and transcriptomic analyses. SSRN.

[37] Ferreti J.D., Ribeiro B., Bonetti J.d.A., Camargo L.E.A., Creste S., Kuramae E.E., Monteiro-Vitorello C.B. (2026). Soil and Genotype Shape the Sugarcane Phytobiome for Enhanced Environmental Adaptation. Environ. Microbiol. Rep..

[38] Douglas G.M., Maffei V.J., Zaneveld J.R., Yurgel S.N., Brown J.R., Taylor C.M., Huttenhower C., Langille M.G.I. (2020). PICRUSt2 for prediction of metagenome functions. Nat. Biotechnol..

[39] Liu M., Xia F., Zhu W., Zhou X., Zhou Z., Zhang J., Su H. (2026). Land Use Change Drives Divergent Assembly and Disassembly Mechanisms in Aquatic Eukaryotic vs. Prokaryotic Microbiomes. Diversity.

[40] Shen Y.F. (2007). Encyclopedia of Liquor Production Technology.

[41] Zhang H., Wang Y., Meng Y., Li A., Zhou Q., Hu X., Liu G., Li L., Huang Y., Xing X.H. (2020). Analysis of Prokaryotic Community Structures in Bottom and Wall Muds from Old and Young Pits Used for Production of Strong-Flavor Baijiu. Food Sci..

[42] Gao J., Ren C., Liu G., Ge X., Wang H., Ye F., Xu Y. (2019). Dynamic changes of flavor compounds and microbial community in fermented grains of Chinese strong aroma-type Baijiu during fermentation. Food Ferment. Ind..

[43] Chen Y., Xie J., Yuan X., Zhang C., Hong J., Zhao Z., Zhao D., Sun B., Wang S., Sun J. (2025). Flavoromics-based profiling of flavor compound dynamics across the strong-flavor Baijiu production chain. J. Food Compos. Anal..

[44] Chen X.M., Lian X.D., Ye S.T., An M.Z., Zhao D., Zheng J., Sun X.T., Dong W. (2024). Accurate Detection of 8 Long-Chain Fatty Acid Ethyl Esters in Baijiu Based on Magnetic Solid Phase Extraction-Gas Chromatography-Mass Spectrometry Combined with Isotope Internal Standards. J. Food Sci. Technol..

[45] Wu W., Choi P., Wang H., Hsieh M., Wu H. (2026). Challenges in ITS-based identification of *Cunninghamella bertholletiae* and Rhizopus microsporus due to intragenomic ITS sequence heterogeneity. Microbiol. Spectr..

[46] Liu J., Su J., Ali A., Wang Z., Zhang R. (2022). Potential of a novel facultative anaerobic denitrifying *Cupriavidus* sp. W12 to remove fluoride and calcium through calcium bioprecipitation. J. Hazard. Mater..

[47] Liu J., Shen X., Chen Z., He Z., Wang J., Yang Y., Zhong X., Xiao C., Li S., Wang S. (2026). Investigating Microbial Community Succession and Function in Fermented Grains (Jiupei) of Nongxiangxing Baijiu Using Metagenomics. Food Sci..

[48] Geng X., Ma Z., Chen E., Wang X., Wang B., Zheng F., Sun J., Sun B., Zhang Y., Li Z. (2025). Interactions between pH and Lactobacillus Drove Esters’ Metabolism during the Fermentation of Laobaigan Baijiu. Food Biosci..

[49] Dong W., Peng Y., Ma J., Hu Y., Chen S., Zhao S. (2025). Yeasts in traditional Baijiu fermentation: Diversity, functions, microbial interactions and applications. Front. Microbiol..

[50] Wang J., Liu Z., Wang X., Zhu J., Xu J., Zhang R., Cai F., Zhu Z., Cao J., Yu Q. (2024). Optimizing Baijiu fermentation with high-yield ethyl caproate-producing *Candida parapsilosis* strain. LWT.

[51] Wu M., Liao H., Luo Y., Yao Y., Yang D., Hu Z., Gao L., Xia X. (2026). Moisture transfer-driven quality enhancement in solid-state fermented Daqu: Synergistic effects of microbial community adaptation and functional enzyme metabolism. Bioresour. Technol..

[52] Wang R., Xu Y., Wang D. (2025). Differences in brewing characteristics between saccharifying microorganisms, Rhizopus and Aspergillus, for the fermentation of rice wine. Food Biosci..

[53] Pu S., Yan S. (2022). Fungal diversity profiles in pit mud samples from Chinese strong-flavour liquor pit. Foods.

[54] Ren H., Li Z., Liu M., Cai Z., Sun Y., Guo X., Fan W., Zhang B., Li Y., Wei J. (2024). High-throughput Sequencing Analysis of Diversity and Spatial Heterogeneity of Fungal Community in Pit Muds of Different Ages for Baijiu Production. Food Sci..

[55] Ye G., Lu L., Xia S., Huang D. (2025). Study on acid-producing metabolic pathway of caproic acid bacteria ZCY20-5 based on whole genomics and transcriptomics. Food Bioprod. Process..

[56] Zhang L., Zheng J., Ren C., Xu Y. (2026). A novel lactic acid bacterium, *Sporolactobacillus* sp. LBM15018, enhances the growth and caproate fermentation capacity of *Caproicibacterium lactatifermentans* through interspecies cross-feeding. Bioresour. Technol..

[57] Puente-Sánchez F., Pascual-García A., Bastolla U., Pedrós-Alió C., Tamames J. (2024). Cross-biome microbial networks reveal functional redundancy and suggest genome reduction through functional complementarity. Commun. Biol..

[58] Wang M., Chen X., Fang Y., Zheng X., Huang T., Nie Y., Wu X. (2024). The trade-off between individual metabolic specialization and versatility determines the metabolic efficiency of microbial communities. Cell Syst..

[59] Zhao C., Yang L., Li H., Deng Z. (2024). Nitrogen metabolism during anaerobic fermentation of actual food waste under different pH conditions. Fermentation.

[60] Adour L., Couriol C., Amrane A. (2005). Diauxic growth of *Penicillium camembertii* on glucose and arginine. Enzym. Microb. Technol..

[61] Deng J., He C., Wei C., Luo H., Huang Z. (2025). Characterization of fermentation parameters, volatile compounds and microbial community structure of fermented grains in the upper and lower layers during strong-flavor Baijiu fermentation. Bioresour. Bioprocess..

[62] Wang J., Lu C., Xu Q., Li Z., Song Y., Zhou S., Zhang T., Luo X. (2022). Bacterial diversity and lactic acid bacteria with high alcohol tolerance in the fermented grains of soy sauce aroma type baijiu in North China. Foods.

[63] Mandlaa M., Liu M., Ren G., Qu H., Wang H., Ma F., Zhang M., Guo J., Chen Z., Sun Z. (2025). Composition and distribution characteristics of acetic acid-related bacteria during the fermentation process of strong-flavor baijiu. Front. Microbiol..

[64] Fan J., Chen J., Du G., Fang F. (2024). Mechanism of lactic acid synthesis during strong-flavor baijiu fermentation revealed by transcriptomic analysis. Food Biosci..

[65] Adeniyi A., Bello I., Mukaila T., Sarker N., Hammed A. (2023). Trends in biological ammonia production. BioTech.

[66] Blachier F. (2025). Amino acid metabolism for bacterial physiology. The Evolutionary Journey of Amino Acids: From the Origin of Life to Human Metabolism.

[67] Zhou Y., Hua J. (2025). Lactic acid dynamics in Baijiu brewing: Microorganisms, roles, and control strategies. Fermentation.

[68] Qiu W., Ru H., Wang J., Kuang J., Yu Y., Zheng Q. (2025). Odor threshold dynamics during Baijiu aging: Ester–acid interactions. LWT.

[69] Li J., Guan X., Huang W., Zhong X., Sun H., Song M., Tang W. (2025). Exploring the influence of moisture stress on microbial-driven organic acid synthesis in potato waste fermentation. Food Chem..

[70] Li X., Tan G., Chen P., Cai K., Dong W., Peng N., Zhao S. (2023). Uncovering acid resistance genes in lactic acid bacteria and impact of non-viable bacteria on bacterial community during Chinese strong-flavor baijiu fermentation. Food Res. Int..

[71] Lin B., Tang J., Li Q., Zhu L., Jiang W., Ke H., Wen Z., Liu H., Yang S., Yang Q. (2025). Bacterial community improves the volatile components coupled with abiotic factors during the spontaneous fermentation of Chinese strong-aroma Baijiu. Food Chem. X.

[72] Chen J., Zhang J., Zeng R., Lin Y., Duan Y., Song C., Zhang S., Zuo Y. (2025). Distribution characteristic, synthesis mechanism and regulation measures of ethyl acetate in strong flavor Baijiu: A review. Food Biosci..

[73] Zhou H., Yan L., Xu B., Zhang L., Wang R., Chen Q., Li X. (2026). Heterogeneous selection driven by pH determines the successional pattern of bacterial communities in pit mud used for baijiu fermentation. Int. J. Food Microbiol..

[74] Gao Z., Wu Z., Zhang W. (2020). Effect of pit mud on bacterial community and aroma components in yellow water and their changes during the fermentation of Chinese strong-flavor liquor. Foods.

[75] Yan C., Chen X., Liu Q., Xu T., Zhang Q., Jin X., Liao B., Chen X., Li X. (2024). Effects of *Lactiplantibacillus plantarum* on metabolites and flavors in synthetic microbiota during baijiu fermentation. Foods.

[76] Song C., Cheng Y., Su Y., Bi C., Leng J., Meng Q., Shan A. (2026). Mechanism driving efficient organic acid synthesis by agricultural waste epiphytic microbiota in anaerobic co-fermentation. Ind. Crops Prod..

[77] Zhang Z., Wang Y., Yao Y., Li Y., Xu X., Hou Q., Hu X., Mei X., Guo Z. (2025). Microbial and flavor dynamics of medium-high temperature Daqu: Regional influences and implications for Daqu quality optimization. Food Res. Int..

[78] Zhang X., Guo X., Li F., Usman S., Zhang Y., Ding Z. (2022). Antioxidant, flavonoid, α-tocopherol, β-carotene, fatty acids, and fermentation profiles of alfalfa silage inoculated with novel *Lactiplantibacillus plantarum* and *Pediococcus acidilactici* strains with high-antioxidant activity. Anim. Feed Sci. Technol..

[79] Zhou Z., Wang X., Duan C., Liu Z., Wang Y., Zhong Y., Hu X., Song Z., Yi J., Wang T. (2024). A synergistic fermentation system of probiotics with low-cost and high butyric acid production: *Lactiplantibacillus plantarum* and *Clostridium tyrobutyricum*. Food Biosci..

[80] Niamah A.K., Al-Fekaiki D.F., Al-Sahlany S.T.G., Verma D.K., Patel A.R., Singh S. (2023). Investigating the Effect of Addition of Probiotic Microorganisms (Bacteria or Yeast) to Yoghurt on the Viability and Volatile Aromatic Profiles. J. Food Meas. Charact..

[81] Tong W., Cheng J., Zhang Y., Cheng W., Chen B., Hu J., Yang Y., Huang D., Luo H., Shen Y. (2026). Exploration of the microbial assembly process of Sauce-flavored Baijiu and the core microorganisms responsible for ester production. LWT.

[82] Shapkin V., Zelenka T., Větrovský T., Kostovčík M., Eichlerováet I., Kohout P., Žifčáková L., Borovička J., Tomšovský M., Adamčík S. (2026). Limitations of Common Molecular Markers in Fungal Biodiversity Analysis and the Benefits of Their Synergistic Use. Mol. Ecol. Resour..

