# Supplementary material for: Differences in Microbial Community Succession and Caproic Acid Metabolic Dynamics in Upper-Layer Fermenting Grains Between Aged and New Pits During Strong-Flavor Baijiu Fermentation
Source: Foods. 2026 Nov 5;15(21):3975. doi: 10.3390/foods15213975 (PMC13762653; doi:10.3390/foods15213975)
Supplement: Supplementary file 1 [file foods-15-03975-s001.zip › Supplementary Figures-20261026.pdf]

Supplementary Figures

Differences in Microbial Community Succession and Caproic Acid Metabolic Dynamics in Upper Fermenting Grains Be-tween Old and New Pits During Strong-Flavor Baijiu Fermen-tation

Huimin Zhang [1]*, Kangjie Xu [1], Qiang Chang [2], Li Zhen [1], Yongqing Li [2], Tianwen Wang [1], Bo Wang [1], and Yan Liu [1]*

[1]College of Biological and Food Engineering, Anhui Polytechnic University, Wuhu, 241000, China

[2]Anhui Jinyuwan Distillery Co., Ltd., Fuyang, 236500, China

*Correspondence: Huimin Zhang: zhm_catharine@126.com; Yan Liu: liuyan@ahpu.edu.cn

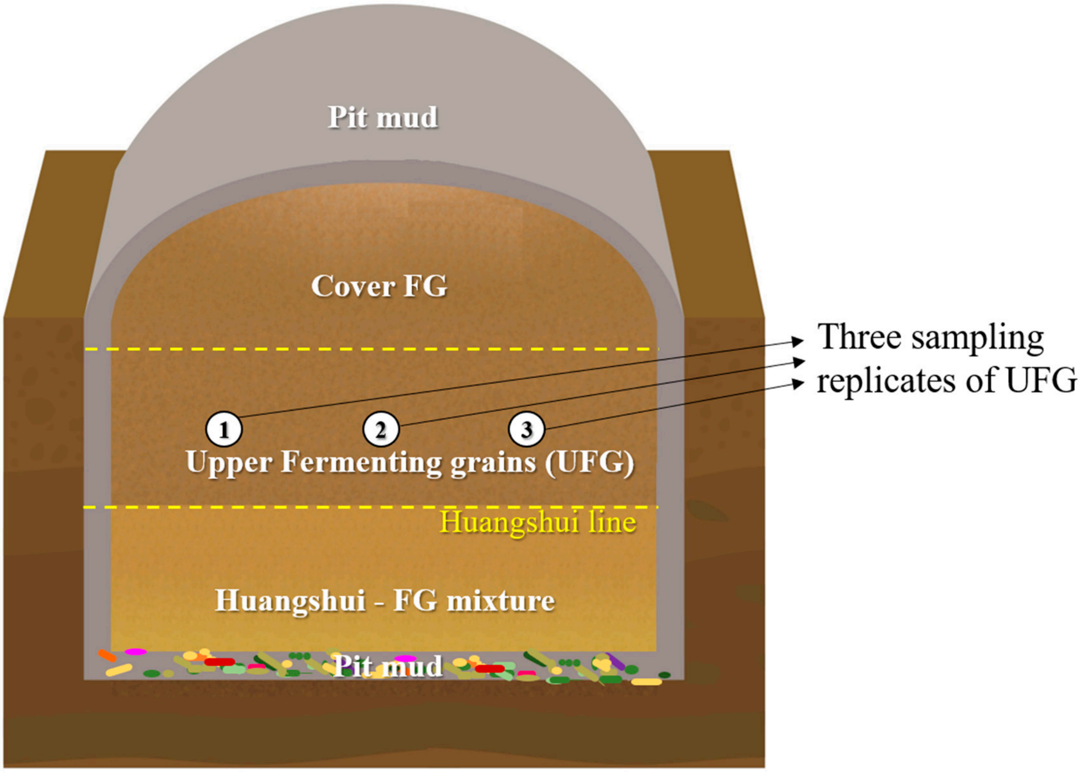

Figure S1 Schematic illustration of the sampling method for upper fermenting grains (UFG). Samples were collected from the interval between the Huangshui line and the covering FG layer.

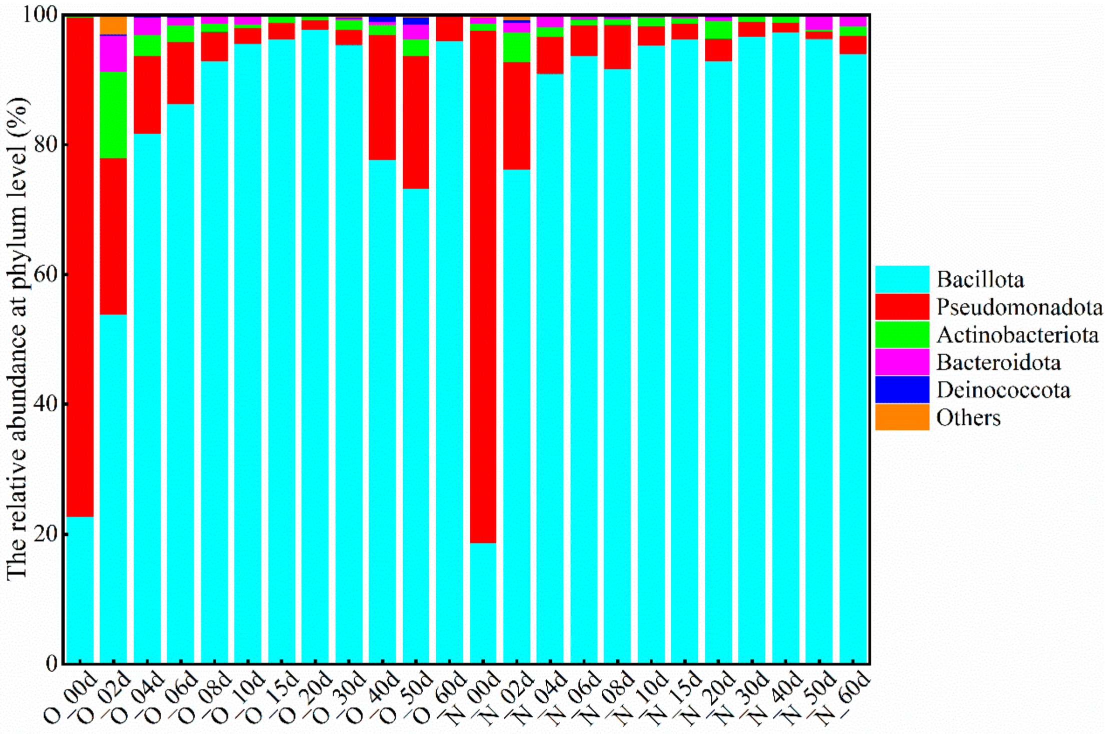

Figure S2. Temporal succession of prokaryotic communities at the phylum level in upper fermented grains (UFG) from new and aged pits during the 60-day fermentation.

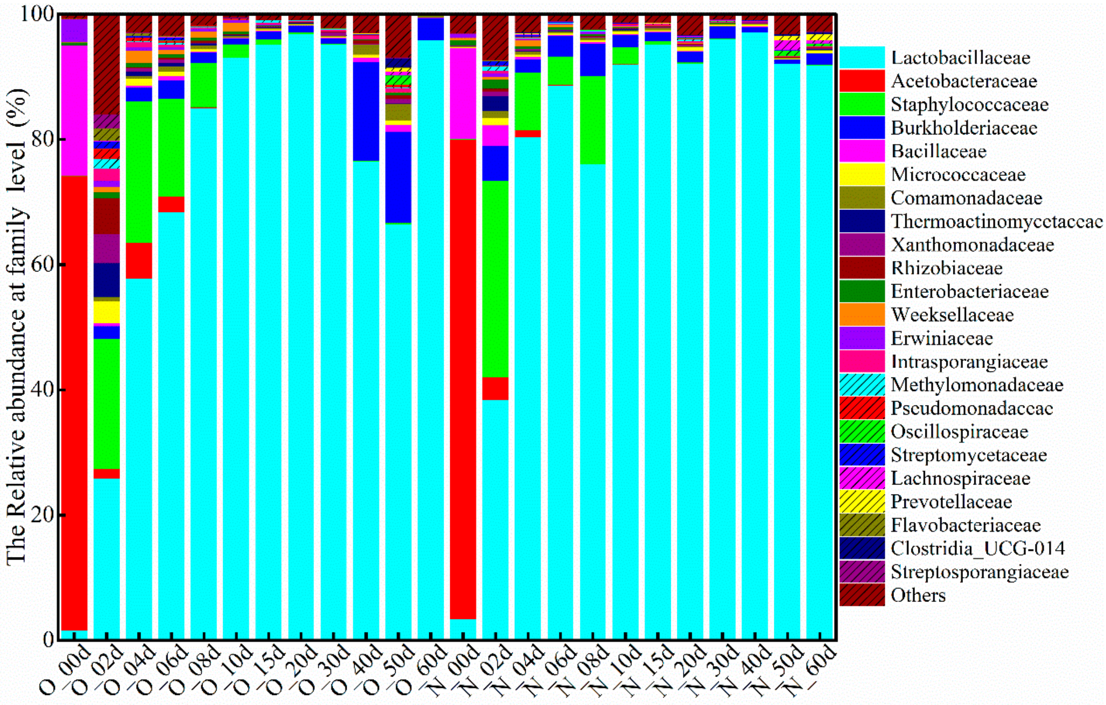

Figure S3. Temporal succession of prokaryotic communities at the family level in upper fermented grains (UFG) from new and aged pits during the 60-day fermentation.

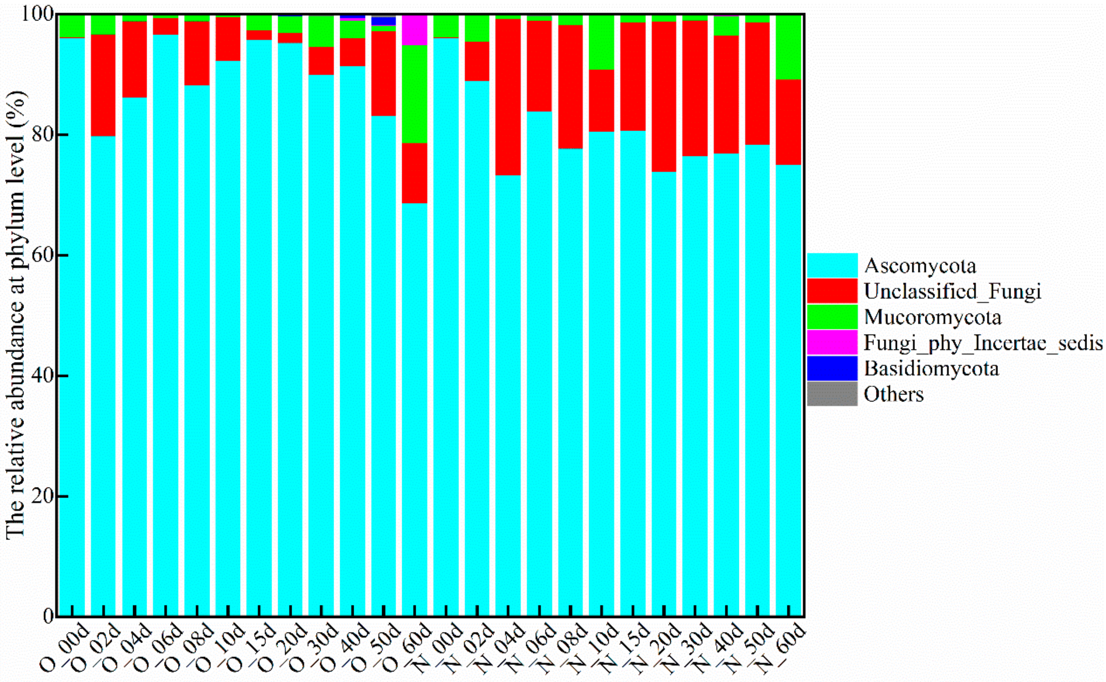

Figure S4. Temporal succession of eukaryotic communities at the phylum level in upper fermented grains (UFG) from new and aged pits during the 60-day fermentation.

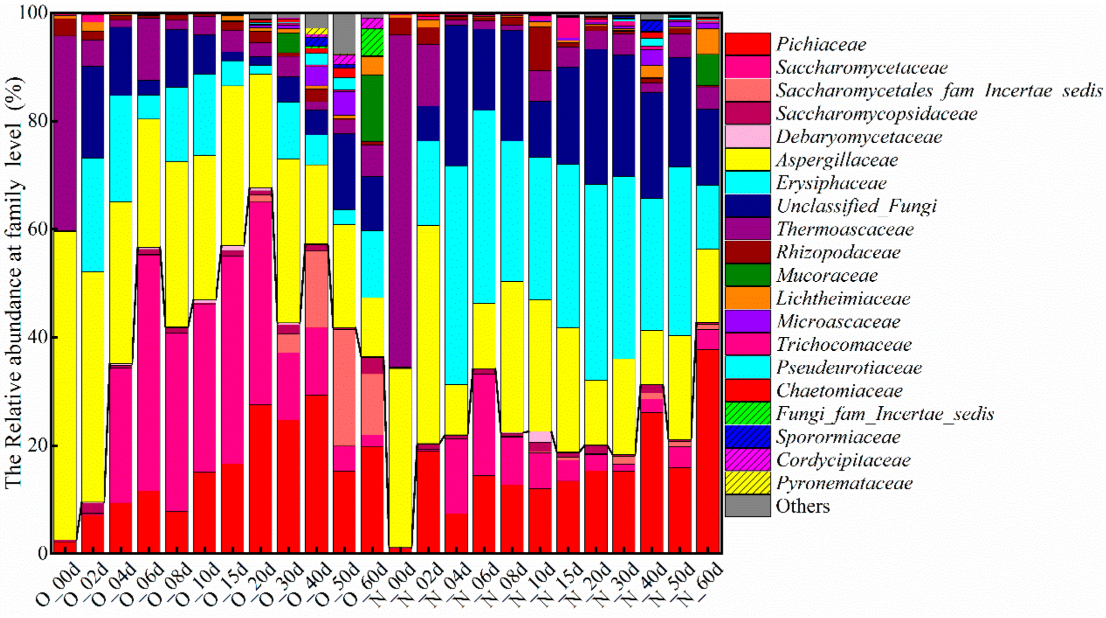

Figure S5. Temporal succession of eukaryotic communities at the family level in upper fermented grains (UFG) from new and aged pits during the 60-day fermentation.

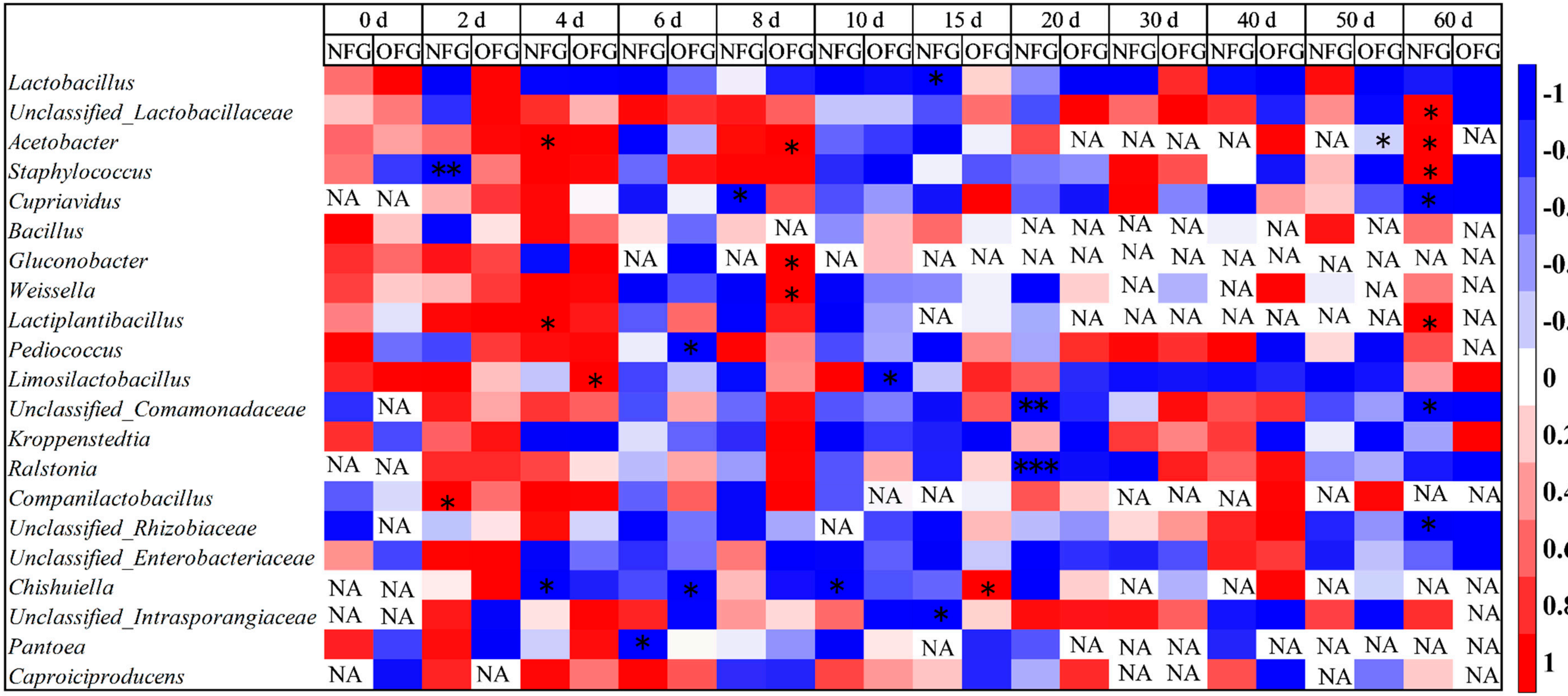

Figure S6. Temporal dynamics of Pearson correlations between PICRUSt2-predicted fatty acid biosynthesis gene abundance and the absolute abundance of dominant prokaryotic genera in new (NFG) and aged-pit UFG (OFG). NA indicates missing correlation coefficients due to zero values in samples.

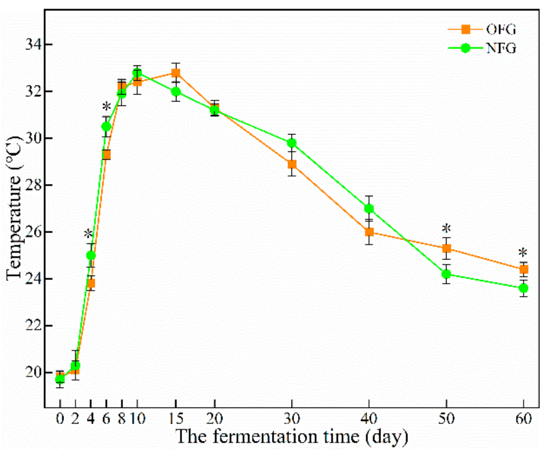

(a)

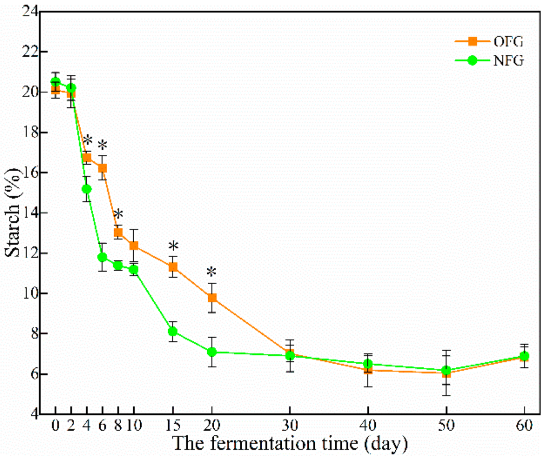

(b)

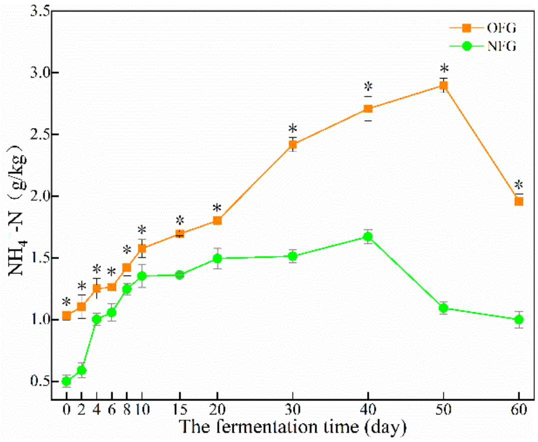

(c)

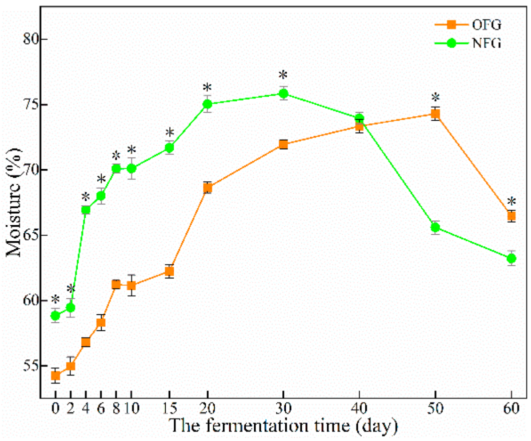

(d)

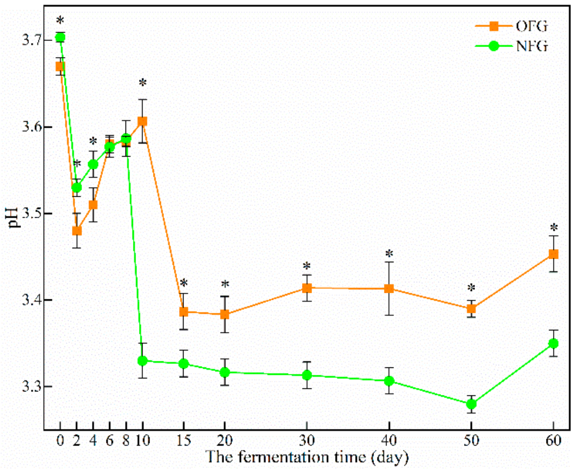

(e)

Figure S7. Dynamics of temperature (a), starch (b), NH_4^+-N(c), moisture (d), and pH (e) of UFG during fermentation from new and aged pits. Data are presented as means ± SD (n = 3 independent pits per group; error bars represent the standard deviation calculated across the three pits). Asterisks indicate significant differences between new and aged pit samples at the corresponding time points ($p < 0.05$, ANOVA).

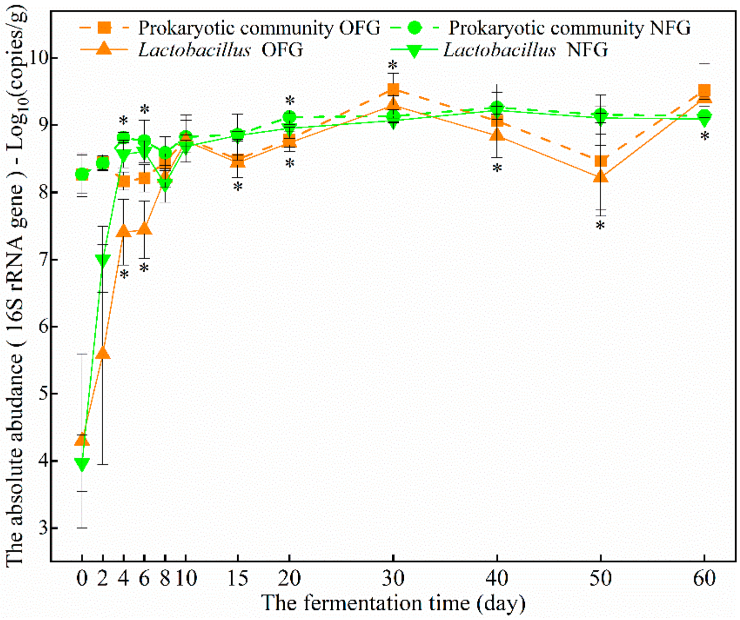

Figure S8. Absolute abundances of the total prokaryotic community and the dominant *Lactobacillus* in UFG from new and aged pits during fermentation. Data are presented as means ± SD (n = 3 independent pits per group). Asterisks above the data points indicate significant differences ($p < 0.05$, ANOVA) between the total 16S rRNA gene copy numbers of the prokaryotic community in new-pit UFG ("Prokaryotic community NFG") and those of the dominant genus *Lactobacillus* in new-pit UFG ("*Lactobacillus* NFG"); asterisks below the data points indicate significant differences ($p < 0.05$, ANOVA) between the total 16S rRNA gene copy numbers of the prokaryotic community in aged-pit UFG ("Prokaryotic community OFG") and those of the dominant genus *Lactobacillus* in aged-pit UFG ("*Lactobacillus* OFG").

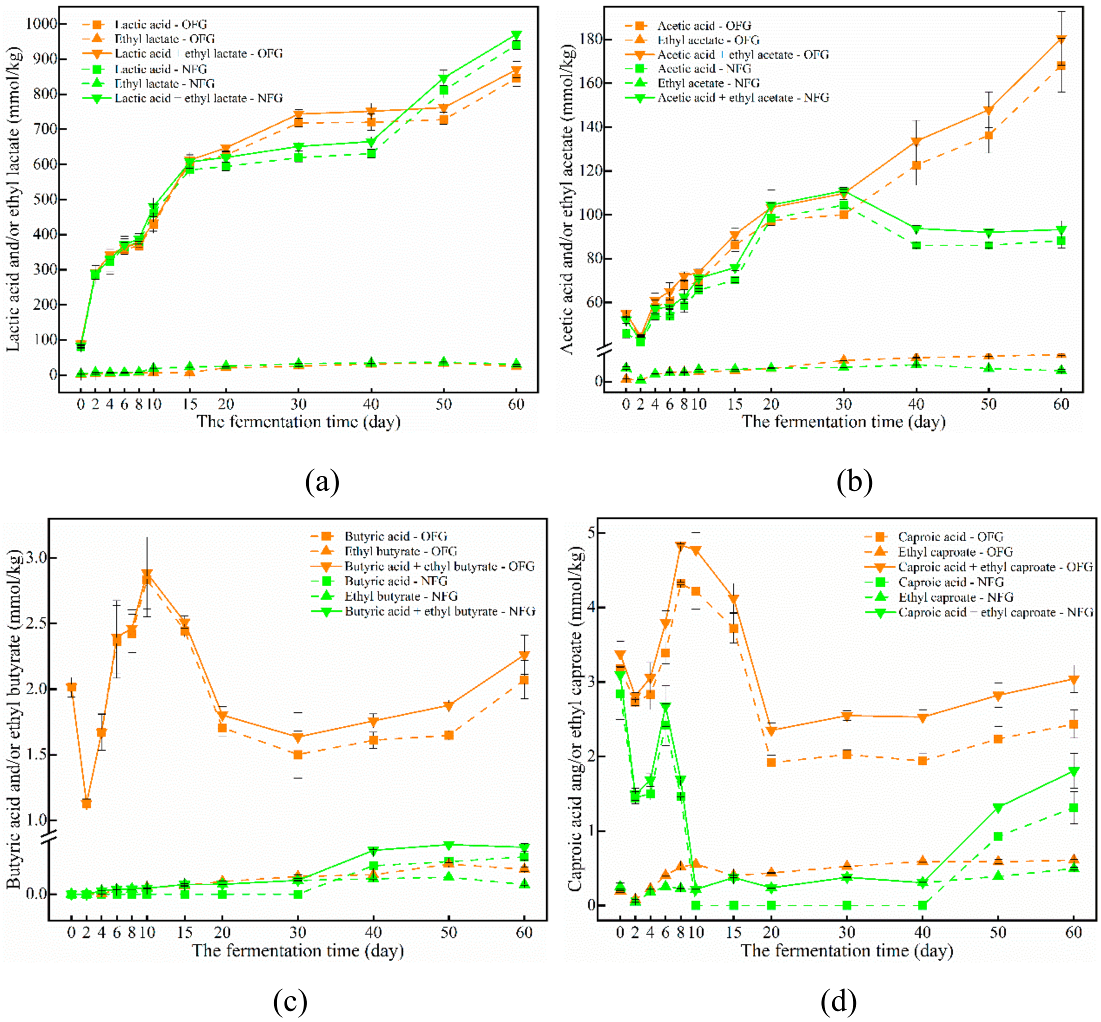

Figure S9. Dynamics of the total molar amounts of the four major acids and their corresponding ethyl esters in UFG from new and aged pits during fermentation. The molar sum of each acid–ester pair was calculated to reflect the total molar quantity of the acid synthesized during fermentation. (a) Lactic acid + ethyl lactate; (b) Acetic acid + ethyl acetate; (c) Butyric acid + ethyl butyrate; (d) Caproic acid + ethyl caproate. Data are presented as means ± SD (n = 3 independent pits per group).

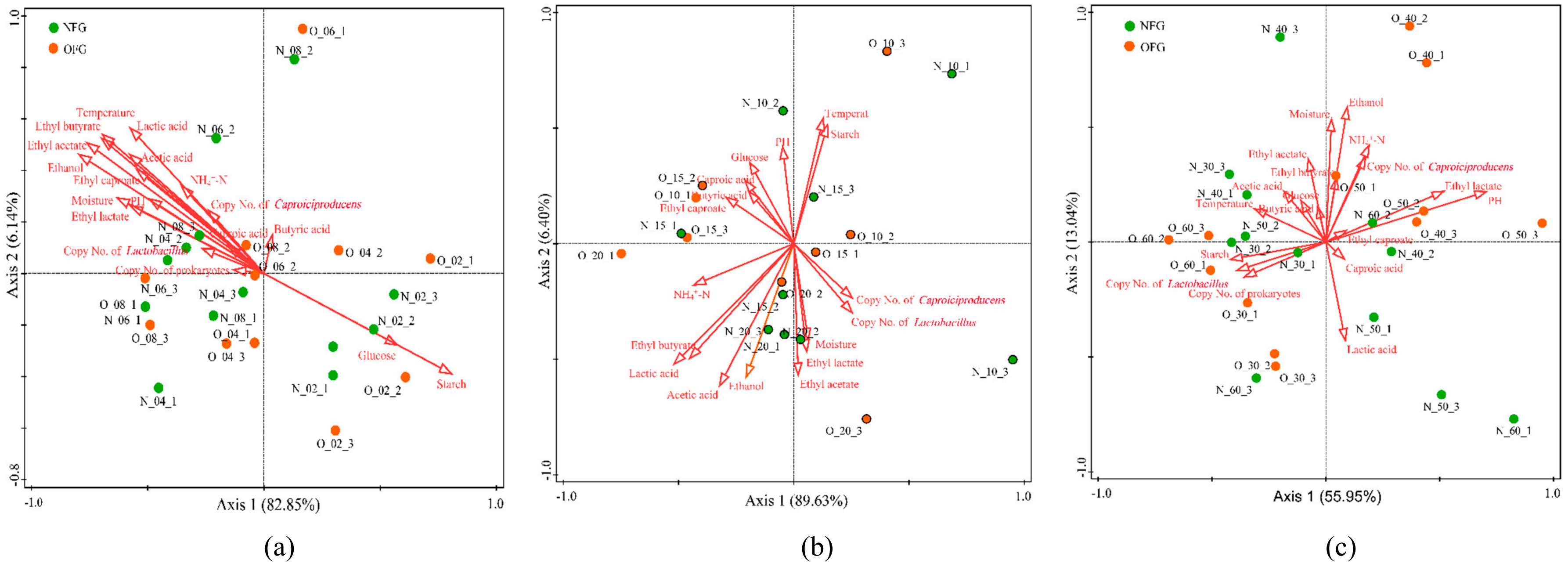

Figure S10. RDA ordination biplots showing the relationships between prokaryotic community structure and physicochemical attributes in new and aged pit UFG across three fermentation stages: 2-8 d (a), 10-20 d (b), and 30-60 d (c). The arrows indicate the direction and magnitude of physicochemical attributes associated with prokaryotic community structures.

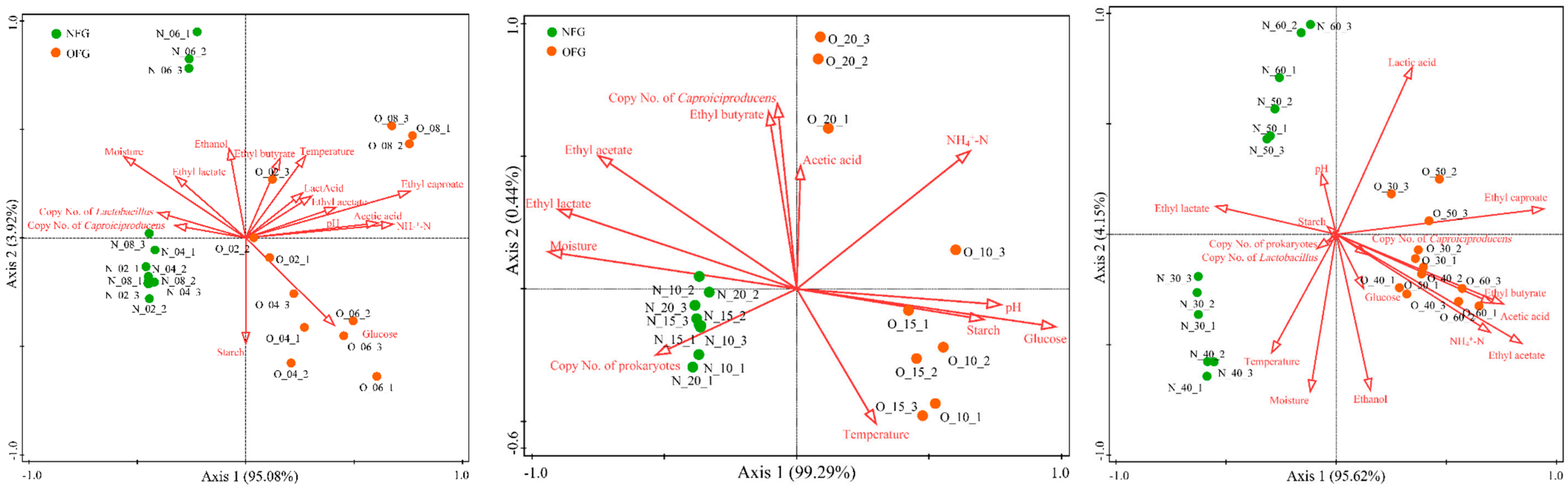

(a) (b) (c)

Figure S11. RDA ordination biplots showing the relationships between physicochemical factors excluding butyric and caproic acids and the accumulation of butyric and caproic acids in new and aged pit UFG across three fermentation stages: 2-8 d (a), 10-20 d (b), and 30-60 d (c). The arrows indicate the direction and magnitude of physicochemical attributes associated with prokaryotic community structures.
